# Development and validation of contextual measures of sexual harassment perceptions, experiences, and coping for women employees in Ethiopian hospitality workplaces

**DOI:** 10.1186/s13690-022-00828-z

**Published:** 2022-02-18

**Authors:** Mulugeta Dile Worke, Zewdie Birhanu Koricha, Gurmesa Tura Debelew

**Affiliations:** 1 Department of Midwifery, College of Health Sciences, Debre Tabor University, Debre Tabor, Ethiopia; 2 Department of Population and Family Health, Faculty of Public Health, Jimma University, Jimma, Ethiopia; 3 Department of Health, Behavior, and Society, Faculty of Public Health, Jimma University, Jimma, Ethiopia

**Keywords:** Sexual harassment, Reproductive health, Scale development, Coping, Validation, Hospitality industry, Ethiopia

## Abstract

**Background:**

Sexual harassment among female employees in the hospitality industry is a complex phenomenon, and it has ramifications for employment, psychological, physical, and reproductive health. Nevertheless, our interpretation is constrained by a lack of agreement on its definition and measurement. As a result, hospitality workplaces require accurate tools that provide a detailed understanding of sexual harassment and inputs for action to limit adverse outcomes. Thus, this study aimed to develop a reliable and valid measure of female hospitality employees’ perceptions, experiences, and coping features concerning sexual harassment.

**Methods:**

Item development, scale development, and scale evaluation were all parts of the design process. Following a round of feedback from the expert group, qualitative results, and a comprehensive literature review on related themes, item pools were created for the first version of the questionnaire. Pre-testing, survey administration, item reduction, and transformation of extracted latent factors of individual items into a unified and measurable construct were also performed. Field testing included five cognitive interviews with women who had experienced sexual harassment, a pre-test study of 30 women, and a survey of 345 women who worked in hospitality workplaces. Finally, tests for dimensionality, reliability, and validity were conducted.

**Results:**

In Bahir Dar, Ethiopia, 345 women working in the hospitality workplaces, with a mean age of 24.31 ± 4.30 years, took part in this study. The robust maximum likelihood estimation approach was used to do confirmatory factor analysis. The model’s stability was determined by calculating the goodness of fit and the factorial invariance. Subsequently, the validity was confirmed. The findings supported the validity and reliability of the questionnaires designed for the target group. Therefore, the questionnaires can be used as research instruments.

**Conclusions:**

The sexual harassment perceptions, experiences and coping scales provide a locally verified method for assessing sexual harassment in Ethiopia by government authorities and local and international non-governmental organisations, which aid in providing necessary services and the evaluation of efforts aimed at improving workplace safety, workplace health, and psychosocial well-being.

**Supplementary Information:**

The online version contains supplementary material available at 10.1186/s13690-022-00828-z.

## Background

In 2017, the #MeToo movement revealed that sexual harassment (SH) was prevalent in hospitality settings, such as restaurants and hotels [1]. Since then, several initiatives, policies, and regulations have been proposed to curb SH [2]. Despite these interventions, laws, and legislation created to prevent SH, it persists as one of the most severe issues in the hospitality industry [3–5], a primarily unreported and widespread issue [6]. It is a pertinent and prevailing issue among female employees [7, 8], resulting in severe adverse effects [9–12]. In addition to its persistence, prevalence, and under-reporting, it is one of the most detrimental and pervasive barriers to occupation accomplishment and fulfilment [13]. Our qualitative findings [14, 15] and other studies have revealed adverse effects on employees’ physical, mental, and reproductive health [16, 17].

Those subjected to SH were more likely to have had multiple sexual partners and were more likely to have contracted sexually transmitted infections (STIs) in their early adulthood [18]. Hospitality workplaces have been identified as HIV hotspots [19], and HIV prevalence is significant among frontline service employees [20]. It got worse when more young women started selling sex in places like bars, nightclubs, karaoke parlours, and massage parlours [21, 22]. Furthermore, their vulnerability to SH and its consequences increases because of factors such as tips [23], being less sensitive to unwanted sexual attention, its pervasiveness [24], lack of awareness creation mechanisms [4], precarious employment [25], and little knowledge of hotel managers [5]. As a result of these multidimensional causes and outcomes, there is a need for more rigorous research, particularly in hospitality workplaces.

However, research in this sector is inconclusive and problematic. The lack of expressive prevalence rate is one of the significant encounters [26]. This lack of a meaningful prevalence rate is mainly because of the numerous and disparate definitions of workplace SH and different methods [26]. The most well-known obstacles include various exposure to SH, single-item measures, and various behavioural assessment scales [26–28]. Moreover, in addition to the infrequent consideration of construct validity, researchers have revealed that such theoretical developments were inadequate [7, 29–31].

Nevertheless, different sexual experience questionnaire versions [4, 23, 32] without additional psychometric evaluation were used [33]. These versions of the sexual experience questionnaire were developed to investigate SH in the military and schools [34, 35]. The contradiction between the variable description and the use of similar measurement techniques results in significant discrepancies in estimated rates, making it impossible to synthesise or compare SH across studies [36]. Likewise, most questionnaires originate from high-income countries, which tend to be more individualistic and capitalistic [37]. Such scales may be lacking in different sociocultural contexts and may lack ecological validity. Many of the included items were also culturally specific and challenging to adapt to other situations, especially hospitality settings in low-and-middle-income countries [26]. These issues show that SH is a sophisticated phenomenon that we do not fully comprehend due to a lack of agreement on definition and measurement. Thus, workplaces require reliable tools that provide a comprehensive understanding of SH and tips for avoiding negative consequences.

In addition, although customers/clients, supervisors, owners, coworkers, and gatekeepers/agents can all be perpetrators [38–41], most studies have been focused on perpetrators who are members of the organisation (supervisors and coworkers) [42] and only a few studies examined customer/guest perpetrators [42–44]. Furthermore, no research has looked into SH from agents. Even if the severity, coping technique, and impact of harassment change depending on the perpetrator’s type, the scale of the problem is cumulative. There have also been no attempts to develop psychometrically sound hospitality industry-specific measures to examine women employee SH perceptions, experiences, and coping strategies.

However, researchers that study SH in the hospitality workplace, such as ourselves, require an accurate, reliable, and valid technique to assess the magnitude and outcomes of SH among women hospitality workplace employees. Hence, a comprehensive, valid, and reliable tool should be available to measure SH perceptions, experiences, and coping in hospitality workplaces, considering female employees’ victimisation by all types of perpetrators.

Thus, this study aimed to develop a reliable and valid technique for measuring SH perceptions, experiences and coping mechanisms in the hospitality workplace. It would also aid as a platform for other researchers, non-governmental organisations (NGOs), and government officials to eliminate SH and its implications for women working in the hospitality industry.

## Methods

The three phases of this study were item development, scale development, and scale evaluation [45]. Furthermore, the nine steps of scale development and validation were followed, including domain(s) identification and item generation, content validity considerations, pre-testing questions, sample and survey administration, item reduction, extraction of latent factors, tests of dimensionality, reliability, and validity [45, 46].

### Study setting

This research was conducted in Bahir Dar, Amhara National Regional State, Northwest Ethiopia, between July 1 and August 30, 2021. The study setting was described in detail elsewhere [47].

#### Item development

In this study phase, domains were identified, items were generated, and content validity was considered.

### Identification of the domains

The domain’s boundaries and dimensions were defined based on the themes identified in our previous qualitative studies [15, 47]. Item generation was made more straightforward, and appropriate questions that fit the identified domain dimensions were identified. The identified domains were the sexual harassment perceptions, sexual harassment experiences and coping in hospitality workplaces (SA 1).

### Item generation

The item pool was created inductively (the generation of items from the responses of individuals using the qualitative data obtained through focus group discussions and individual women interviews) and deductively (through the description of the identified domains and the identification of items-through literature review and assessment of existing scales and indicators of the identified domains), using data from prior qualitative studies within this population [15, 47] and a thorough examination of published articles on related themes. It was created by evaluating the themes from interviews and focus group discussions with women working in hospitality workplaces in the earlier qualitative findings and a review of related articles published in the literature. The qualitative findings paved the way for creating a practical assessment tool to identify SH-related behaviours among women in hospitality workplaces. Themes from the qualitative data were used to create similar constructs that served as the basis for developing scale items. Then, the item pool’s first draft was created. The review of literature gives a theoretical basis for defining the domains. According to DeVellis’ [48] recommendations, all statements were written to be straightforward, understandable, and unambiguous to the respondents. We wrote the items positively to minimise the possibility of set responses to questions. The response categories were a Likert scale, with five options (i.e., strongly disagree, disagree, neutral, agree, strongly agree) for SH perceptions, and (never, once/twice, sometimes, often, and always) for SH experiences and coping with SH. This process formed the first draft of the scales, named women’s SH perceptions questionnaire for hospitality workplaces (PSHQ_HW), women’s SH experiences questionnaire for hospitality workplaces (SEQ_HW), and women’s SH coping mechanisms for hospitality workplaces (SHCQ_HW), with 32, 78, and 86 items, respectively.

### Forward and back-translations

Two translators worked on the initial version of the questionnaire, written in British English and translated into Amharic (local language). One of the translators was a public health expert, while the other was an Amharic language expert. The translators were fluent in Amharic and had an excellent command of the English language. A third person fluent in Amharic and English looked through the translated version and combined them. Both translators approved the synthesised version.

After that, two different persons back-translated the synthesized version. One was a health practitioner, while the other was not. One of the back-translators held a PhD in English, while the other was an Ethiopian citizen who had spent many years living and pursuing his PhD in Australia. The same individual who synthesized the translated versions examined the two back translations. After that, the two back-translated versions were combined into one. The differently back-translated words were noted and debated. The term was inserted into the synthesized version once an agreement was achieved.

### Expert consensus

Finally, a committee of experts compared the original item pool, the translated version, and the back-translated version. Members of the experts’ panel chosen for their workplace sexual violence and harassment expertise validated the item pool. The experts were five in number and included one psychologist, one language specialist, two reproductive health specialists, and one professional in health, behaviour, and society. Two of them were PhD in qualification, and three were masters of science and master of public health. These experts evaluated each item constituting the domain for content relevance, representativeness, and technical quality. They used the clarity of expression, face validity, appropriateness for the construct being measured, and potential for differentiating the target population as a criterion to judge items for retention or deletion [48]. Each expert scored for each item and commented on the modified items. Things that were regularly judged to be omitted were removed. At this stage, we produced the second draft of the items containing 20 PSHQ_HW, 33 SEQ_HW, and 32 SHCQ_HW items. The remaining items were used to create a questionnaire draft, then submitted to two co-authors (GT and ZB) for face and material validation. All experts and the two co-authors provided feedback and recommendations about whether such elements should be added, removed, or changed. The inter-rater agreement; content validity index for items; scale-level content validity index, universal agreement calculation; averaging calculation method, and modified kappa were computed to indicate the content validity. Items were maintained after the item evaluation based on the five-member experts’ recommendations, and some items were updated to be more readable and explicit (SA 2. A, B & C).

In addition, three rounds of content expert feedback were obtained using the Delphi Approach. In-depth interviews with our target population were interwoven throughout the three rounds. The questionnaires became more refined as each round progressed. Until there was agreement on the description of the domains, we were researching the potential items we could use.

### Cognitive interview

The complete list of items derived from the expert consensus meeting was tested using cognitive interviewing [49, 50] in a sample of women hospitality workplace employees in two rounds to evaluate each item constituting the domain for representativeness of experience from the target population. In the first round, women hospitality workplace employees were recruited consecutively from Bahir Dar city hospitality workplaces. They were asked what they thought each question was asking, whether they could paraphrase each question in their terms, and the logic behind their responses during cognitive interviewing [51] (SA 3). Problems with understanding, the need for clarifications, and any terms or phrases considered inappropriate, insulting, or sensitive were all noted simultaneously. Poorly understood items that were not rephrased and sensitive or unacceptable to respondents were excluded. In the second round, the cognitive interview was conducted to assess the extent to which questions reflect the domain of interest and those answers produce valid measurements. We administered draft questions to 5 interviewees in the second round. We allowed respondents to verbalise the mental process entailed in providing answers. At this stage, we produced the fourth draft of the items containing 17 PSHQ_HW, 29 SEQ_HW, and 27 SHCQ_HW items (SA 4. A & B).

#### Scale development

In this phase, individual items were turned into a harmonious and measuring construct through pre-testing questions, sampling and survey administration, item reduction, and extraction of latent factors.

### Pre-testing

To examine the extent to which the questions reflect the domains being studied and the extent to which answers to the questions produced valid measurements, the last version of the questionnaires was given to a convenience sample of 30 women working in the hospitality industry. First, women’s comments on item clarity and items wording were taken. Then, items were checked for readability, explicitly and accurately, reflecting the intended dimensions of behaviours among women hospitality workplace employees.

### Sampling

Lastly, 348 women hospitality workplace employees were taken based on a participant-to-item ratio of 1:10 and a 20% non-response rate [52, 53]. Cisgender women were selected systematically from each hospitality workplace in Bahir Dar city, and those older than 18 were included. As it was defined in our previous qualitative study [47], the hospitality workplaces included hotels, bars, fast-food restaurants, restaurants, and cafeterias. Women who have been working in these workplaces were selected randomly using the registers of each hospitality workplaces employee list.

### Data collection

**Fig. 1:**
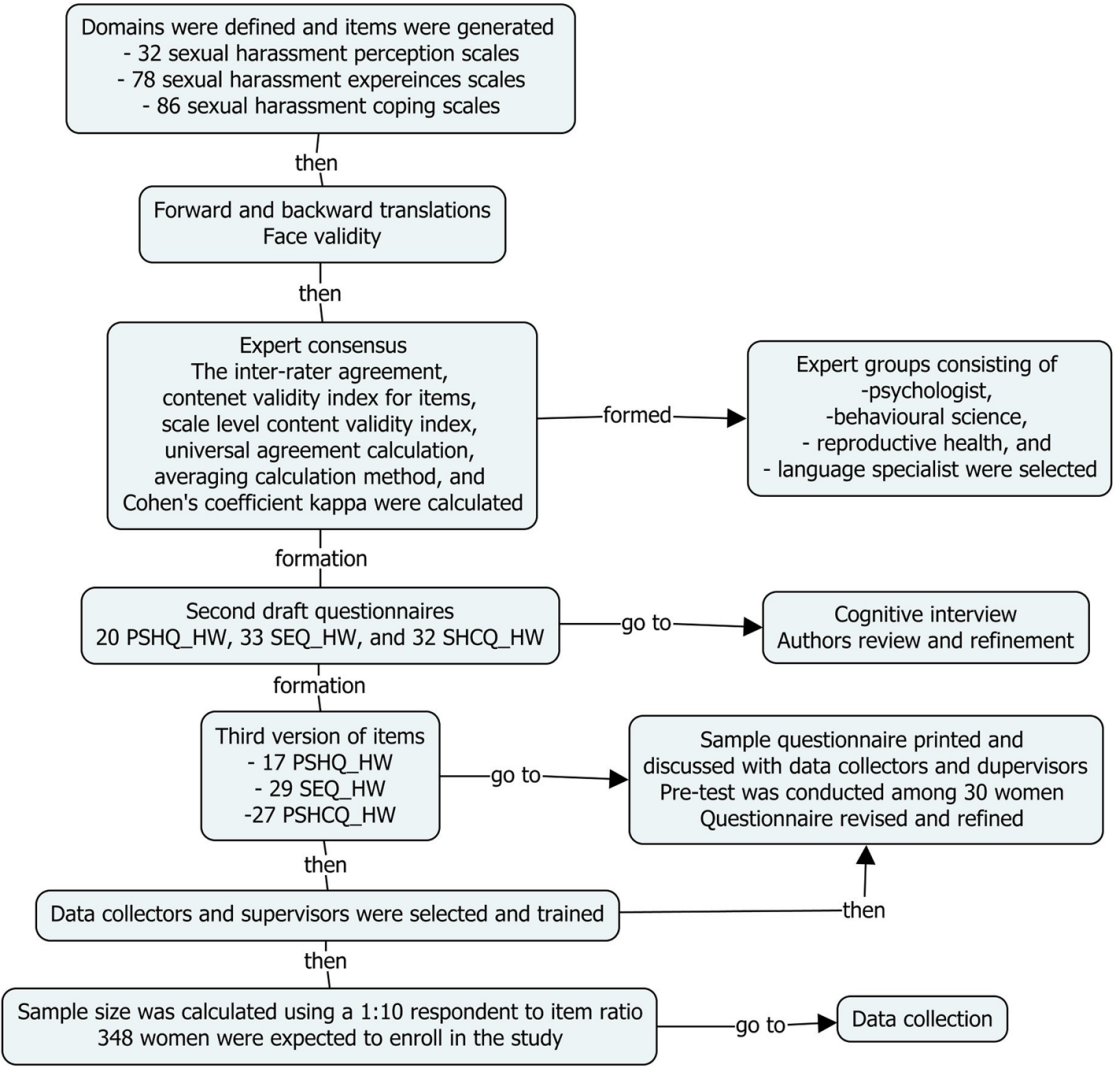
A flowchart of the sexual harassment perception, experiences and coping mechanisms questionnaires development and validation process in Bahir Dar city hospitality workplaces between July 1 and August 30, 2021

Trained data collectors with experience in data collection on related topics were recruited from Bahir Dar University. The data collectors and supervisors received a two-day intensive training on identifying the women, collecting the data, and overseeing to assure data quality. The data collectors gathered the data using the developed questionnaires at hospitality workplaces in Bahir Dar, our previous qualitative analysis location. The survey was completely voluntary and anonymous. Activities such as intense supervision at the field and daily revision of the collected questionnaire were conducted to ensure the collected data’s quality. Since the questionnaire was self-administered in hospitality workplaces, where most female employees cannot read and write a simple sentence, they may face difficulty realising the difference between one item and another. In such conditions, data collectors made their maximum effort to encourage the respondents to ask for any ambiguity in the items and clarify them accordingly. As a result, further clarification was done in any deviation from the tool’s correct material (Fig. 1). After the appropriate coding by the principal investigator, the data were entered using the Epi Info version 7 software in a well-prepared data entry template. After the entrance, the data were rechecked its correctness, screened for missing, outlier values and data entry errors using the frequency distribution of the variables and observation of the entered data. Actual and suspected errors were validated against the raw data, and corrections were made.

### Data analysis

Statistical Package for the Social Sciences (SPSS, version 24.0 (SPSS, IBM, Armonk, NY, USA)), Analysis of Moment Structure (AMOS, IBM, Armonk, NY, USA), version 23.0), and jamovi version 2.0.0 for windows were used to conduct the statistical analyses. Consequently, the data were analysed for face and content validity, reliability, dimensions, and correlations. In addition, frequency, percentage, mean, and standard deviation were used to examine the distribution of responses to each item. Items with low mean values compared to other items and those supported or rejected by most respondents were examined for adjustment or deletion.

### Item reduction analysis

Item reduction analysis was conducted to ensure only parsimonious, functional, and internally consistent items were included in the analysis. Items with exceptionally low item-scale correlations (R ≥ 0.9) [54] were either deleted or merged. Exploratory factor analysis was used to identify potential dimensions under the sub-scales that emerged from the qualitative studies and items that load on each of these dimensions. Items with factor loadings lower than 0.5 and items with cross-loadings (> 0.40) were deleted. At least 0.5 variances were explained by the variable to retain in the model.

### Extraction of factors

We used a scree plot and variance explained by the factor model, eigenvalue of > 1, and the pattern of factor loadings to determine the number of factors retained. Exploratory factor analysis (EFA) was used to determine the optimal number of items that fit a set of items. Kaiser–Meyer–Olkin (KMO) values were used to assess sampling adequacy, and Bartlett’s test of sphericity (preferably significant) was used to assess data suitability for factorisation.

The EFA was used to explore the common factors in the latent variable using SPSS version 24.0. The maximum likelihood was used to decide the number of factors to be extracted, and the Promax with Kaiser normalization was the rotation method used to identify essential components. The criterion for loading and cross-loading was set at 0.4. As a result, items with loading less than 0.5 and cross-loading greater than 0.4 were removed. This process was repeated until a straightforward structure was achieved where loadings were maximised on putative factors and minimised on the others [55–57]. At least three variables per factor were needed to identify stable factors [58].

#### Scale evaluation

At this phase, tests of dimensionality, reliability tests, and validity tests were conducted. The questionnaire’s tests of dimensionality were cross-validated using Confirmatory Factor Analysis (CFA). The CFA model’s goodness of fit was measured using several model fit indices. The indices which were utilised in this study were the root mean square error of approximation (RMSEA), comparative fit index (CFI), standardised root mean square residual (SRMR), incremental fit index (IFI), CMIN (Chi-square), Comparative Fit Index (CFI), Tucker–Lewis’s index (TLI), and PGFI (Parsimony-adjusted Goodness of Fit Index) [59].

Reliability statistics was conducted to assess the internal consistency of the scale, i.e., the degree to which the set of items in the scale co-vary, relative to their sum score. An estimate was conducted using Cronbach’s alpha coefficients, composite reliability (CR), and Maximum reliability (MaxR(H)).

### Tests of validity

#### Criterion validity

Predictive validity was conducted to determine if the scores predict future outcomes, and concurrent validity was used to determine the extent to which scale scores have a stronger relationship with criterion measurements made near the time of administration. Bivariate and multivariate regressions were used to estimate the predictive validity, and Pearson product-moment correlation was used for concurrent validity.

#### Construct validity

At this phase, convergent validity and discriminant validity were used to determine if the same concept measured in diverse ways yields comparable results and if the concept measured is different from some other concepts. The relationship between scale scores and similar constructs was estimated using Composite Reliability (CR), Average Variance Extracted (AVE), Maximum Shared Variance (MSV), and Average Shared Variance (ASV) [60, 61].

## Results

### Sociodemographic characteristics

Three hundred and forty-five women hospitality employees were participated in this study, making a response rate of 99.13%. Hundred and five (30.4%) were married at a mean age and standard deviation (SD) of 19.57 (± 3.34) years. The mean age and SD of the sample was 24.31 (± 4.30) years. Women employees’ average distance and SD travelled 5.56 (± 9.84) kilometres.

The respondents had 1.58 (± 0.51) years of experience, 1498.26 (± 945) Ethiopian birrs monthly salary and 1997.07 (± 1239.40) monthly income, including tips. They were also at work for 10.10 (± 2.63) hours per day (SA 5).

### Exploratory factor analysis and scale reduction

The 17 items of the SH perceptions scale, 29 items of the SH experiences scale, and 27 items of SH coping techniques scale were initially subjected to principal component analysis to determine the optimum number of factors to parsimoniously explain the order and structure of the scale’s items. Three factors with an eigenvalue greater than 1.0 [62] were kept in each scale. The maximum likelihood of the factor loading matrix was used to assess solutions for one or three factors. A total of 67.6, 58, and 55.4% of the variances were explained by the 3-factor solutions for SH perceptions, experiences, and coping, respectively. The scree plot was investigated further to discover significant factors. Cattell’s scree test (i.e., levelling of eigenvalues following an “elbow”) confirmed the appropriateness of the three factors solution in each scale. These were also supported by the findings of a parallel analysis [62]. This analysis compared against each eigenvalue to the eigenvalue for the corresponding factor in 1000 randomly generated datasets with similar features to the sample being analysed. The oblique rotation (Promax with the default Kappa 4) was employed to achieve rotated factor loadings because of high correlations between 3 factors (r ≥ 0.5) in each scale. Only individual items with primary loadings of 0.4 or higher and no cross-loading of 0.3 and higher were kept for each factor extracted. Cronbach’s alpha coefficient was used to determine the scale’s internal consistency. Cronbach’s Alpha of 0.70 or higher was regarded as satisfactory. Additionally, item-item bivariate correlations were evaluated to ensure that items with exceptionally low (r < 0.5) correlations were deleted.

**Fig. 2:**
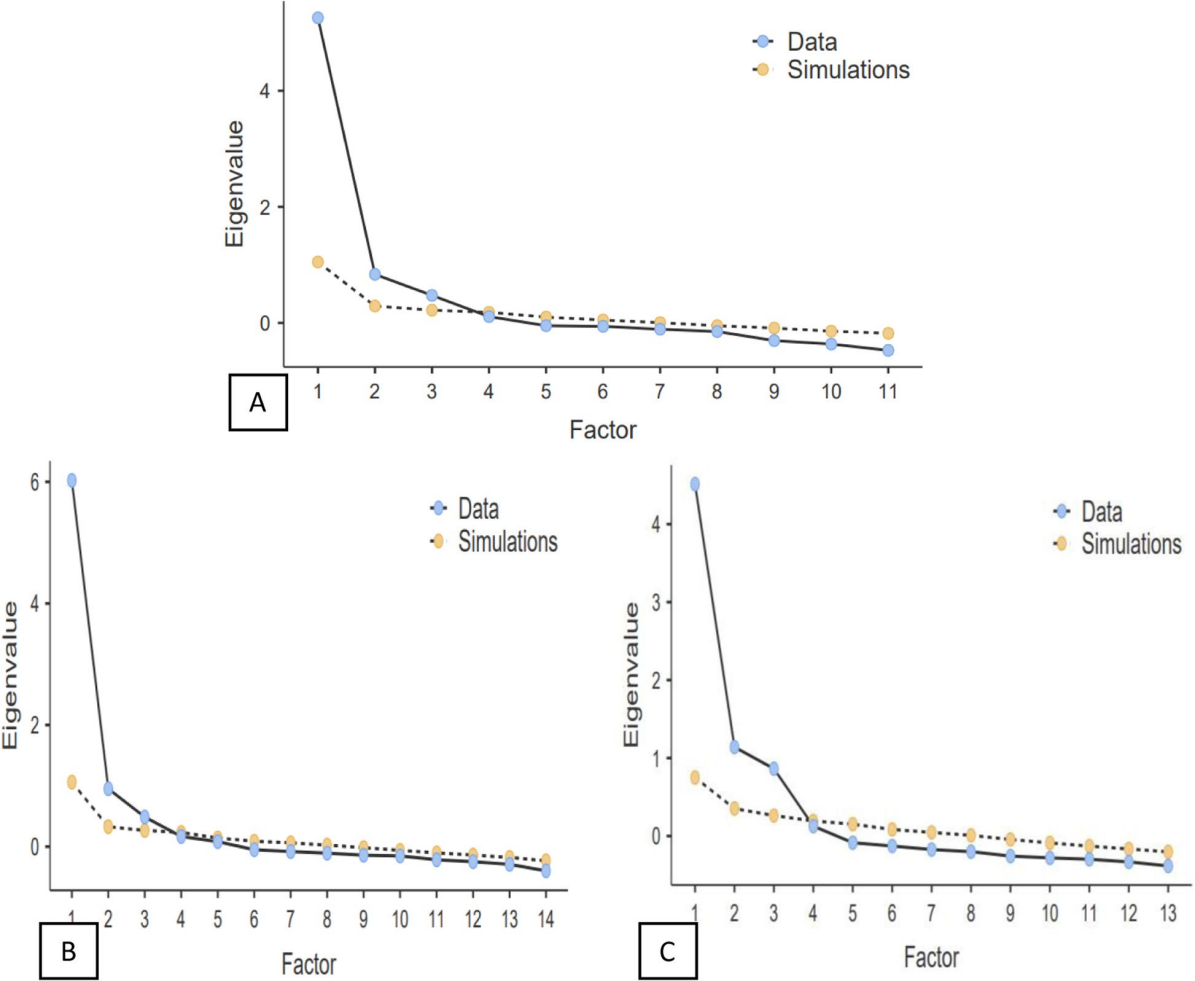
The Scree plots **A**) Sexual harassment perception, **B**) Sexual harassment experiences, and **C**) Coping with sexual harassment among women working in Bahir Dar city hospitality workplaces between July 1 and August 30, 2021

The extraction of the three factors was supported by EFA, the Kaiser criterion, the resulting scree plot elbow point and parallel analysis of the 17-item SH perceptions scale. Three items (i.e., 12, 13, 14) were eliminated using EFA with a Promax rotation for a three-factor solution. They all had a loading value of less than 0.4. Three items (i.e., items 9, 10, and 11) loaded the least and caused convergent and discriminant validity issues, so they were removed from the model after further analysis using CFA and convergent and discriminant validity. Finally, an 11-item perceptions scale of SH was retained (Fig. 2). The EFA, CFA, and discriminant and convergent validity analysis results are presented (Table 1).

**Table 1:** Factors, loadings, the Cronbach’s Alpha if item deleted, and Cronbach’s alpha coefficients of the extracted sexual harassment perceptions questionnaire among women working in Bahir Dar city hospitality workplaces between July 1 and August 30, 2021

Items		Factors			Cronbach’s Alpha if item deleted
Code	Name	Pressuring	Abducting	Touching	
PSHQ_HW3	Sexual harassment is an offer of a new job in exchange for sexual advances	.984			.901
PSHQ_HW2	Sexual harassment is preparing women for sexual harassment by providing better career advancement	.897			.900
PSHQ_HW1	Sexual harassment is preparing for sexual harassment by making inappropriate promises	.724			.905
PSHQ_HW4	Sexual harassment is an act of threatening to hurt relatives in exchange for sexual advances	.722			.905
PSHQ_HW5	Sexual harassment is threatening to complain or falsely accuse about the service being provided to the immediate supervisor unless women agree to sexual favours	.534			.904
PSHQ_HW16	Sexual harassment is conducting sexual intercourse through force or under threat of injury against the women will		.956		.907
PSHQ_HW17	Sexual harassment is slapping, kicking, pinching, or insulting women while they refuse to agree to a sexual favour		.835		.906
PSHQ_HW15	Sexual harassment is a forcible take of women for sexual intercourse after they leave their job		.757		.910
PSHQ_HW7	Sexual harassment is an act of speaking random sexual jokes to women while she is at work			.952	.907
PSHQ_HW8	Sexual harassment is a repeated request of women to engage in sexual activities			.735	.908
PSHQ_HW6	Sexual harassment is an act of touching sexual sensitive parts while the women are at work			.543	.902
Cronbach’s alpha coefficient		**.898**	**.880**	**.834**	**.913**

The identified factors explained 67.6% of the variance. Bartlett’s test of sphericity was significant (χ2 = 2477.24, df = 55, *p* < 0.001), and Kaiser-Meyer-Olkin measure was 0.89. Cronbach’s Alpha was used to analyse the internal reliability of the final SH perception scale and three subscales. Cronbach’s Alpha was > 0.7 for all subscales, indicating good internal consistency (Table 1). Cronbach’s Alpha for the final SH perception scale was 0.91, and deleting more items did not significantly increase Alpha.

Similarly, three factors were extracted by EFA, the Kaiser criterion, the resulting scree plot elbow point and parallel analysis of the 29-item SH experiences scale. Twelve items were eliminated using EFA with the Promax rotation method for a three-factor solution. All eliminated items had a loading value of less than 0.4. Moreover, the three items (i.e., items 19, 20, and 21) loaded the least and caused convergent and discriminant validity issues, so they were removed from the model after further analysis using CFA and convergent and discriminant validity. Finally, a 14-item SH experiences scale was retained. The EFA, CFA, and discriminant and convergent validity analysis results are presented (Table 2).

**Table 2:** Factors, loadings, the Cronbach’s Alpha if item deleted, and Cronbach’s alpha coefficients of the extracted sexual harassment experiences questionnaire among women working in Bahir Dar city hospitality workplaces between July 1 and August 30, 2021

Items		Factor			Cronbach’s Alpha if item deleted
Code	Name	Verbal	Non-verbal	Physical	
SEQ_HW10	How often do perpetrators target you for rumours of sexual promiscuity?	.845			.905
SEQ_HW9	How often does a perpetrator violate your boundaries?	.814			.903
SEQ_HW8	How often does a perpetrator touch you in a way that makes you feel uncomfortable?	.771			.904
SEQ_HW5	How often do perpetrators have unwanted sexual conversations with you?	.715			.905
SEQ_HW4	How often does a perpetrator sexually assault you in public or in private?	.638			.908
SEQ_HW11	How often do perpetrators insult you by targeting your sexual orientation?	.600			.908
SEQ_HW29	How often do perpetrators make unwanted attempts to stroke, fondle, or kiss you?		.851		.907
SEQ_HW27	How often do perpetrators make unwanted attempts to establish a romantic sexual relationship with you?		.757		.904
SEQ_HW26	How often do perpetrators make you afraid that you would be handled by him poorly if you did not cooperate sexually?		.737		.902
SEQ_HW24	How often do perpetrators make you feel you were being bribed with some reward to engage in sexual behaviour?		.688		.905
SEQ_HW23	How often do perpetrators gaze, leer, or ogle at you in a way that makes you feel uncomfortable?		.670		.905
SEQ_HW17	How often do perpetrators unnecessarily expose themselves in front of you?			1.072	.911
SEQ_HW16	How often does a perpetrator make sexual assaults, attempts of rape, or actual rape?			.474	.910
SEQ_HW18	How often do perpetrators threaten you by filing a complaint about your service to your supervisor because you refused a sexual request?			.404	.906
Cronbach’s alpha coefficient		**.879**	**.872**	**.769**	**.912**

These identified three factors explained 58% of the variability. Bartlett’s test of sphericity was significant (χ2 = 2528.32, df = 91, *p* < 0.001), and the Kaiser-Meyer-Olkin measure was 0.90. Cronbach’s Alpha was used to analyse the internal reliability of the final SH perception scale and three subscales. Cronbach’s Alpha was > 0.7 for all subscales, indicating good internal consistency (Table 2). Cronbach’s Alpha for the final SH perception scale was 0.91, and deleting more items did not significantly increase Alpha.

Furthermore, three factors were extracted by EFA, the Kaiser criterion, the resulting scree plot elbow point and parallel analysis of the 27-item SH coping scale. Ten items were eliminated using EFA with the Promax rotation method for a three-factor solution, and three items (i.e., items 14, 16, and 27) were eliminated because of the low loading (< 0.5) on each factor dimension of EFA. All eliminated items had a loading value of less than 0.4. One item (i.e., item 15) loaded the least and caused convergent and discriminant validity issues, so it was removed from the model after further analysis using CFA and convergent and discriminant validity. Finally, 13-item SH coping scale was retained. The EFA, CFA, and discriminant and convergent validity analysis results are presented (Table 3).

**Table 3:** Factors, loadings, the Cronbach’s Alpha if item deleted, and Cronbach’s alpha coefficients of the extracted sexual harassment coping questionnaire among women working in Bahir Dar city hospitality workplaces between July 1 and August 30, 2021

Items		Factor			Cronbach’s Alpha if item deleted
Code	Name	Normalisation	Engagement	Help-seeking	
SHCQ_HW25	How often did you keep silent to respond to sexual harassment?	.893			.859
SHCQ_HW24	How often did you tolerate sexual harassment?	.740			.857
SHCQ_HW23	How often did you confront the perpetrator?	.696			.860
SHCQ_HW26	How often did you ignore sexual harassment?	.674			.862
SHCQ_HW22	How often did you reject the request for sexual harassment?	.590			.866
SHCQ_HW19	How often did you consult a psychologist because of the sexual harassment that you face?		.921		.861
SHCQ_HW20	How often did you negotiate with the perpetrator?		.773		.861
SHCQ_HW18	How often did you consult a health care provider because of sexual harassment?		.765		.863
SHCQ_HW21	How often did you discriminate against the perpetrators?		.495		.865
SHCQ_HW9	How often did you get sympathy and understanding from friends who have had the same problem?			.739	.861
SHCQ_HW11	How often did you seek reassurance from those who know you best?			.736	.867
SHCQ_HW10	How often did you talk to people about the situation because talking about it makes you feel better?			.686	.863
SHCQ_HW8	How often did you go to a friend for advice on how to change sexual harassment?			.659	.863
Cronbach’s alpha coefficient		**.846**	**.835**	**.811**	**.871**

Cronbach’s Alpha was used to analyse the internal reliability of the final SH coping scale and three subscales. Three factors were extracted and explained 55.4% of the variability. Bartlett’s test of sphericity was significant (χ2 = 1970.30, df = 78, *p* < 0.001), and the Kaiser-Meyer-Olkin measure was 0.87. Cronbach’s Alpha for the entire questionnaire was 0.87, indicating the reliability of the questionnaire items (Cronbach’s alpha coefficient for the three factors was > 0.7, indicating acceptable reliability) (Table 3). Cronbach’s Alpha for the final SH perception scale was 0.91, and deleting more items did not significantly increase Alpha.

### Confirmatory factor analysis

Structural equation modelling was used to test the measurement models. The goodness of fit, composite reliability, convergent, and discriminant validity were evaluated using IBM SPSS AMOS 23. The measurement models determine the link between the latent variable and the observed measurements and whether the data fit the model well [63]. When fit indices are in the marginal ranges, it is essential to consider the model’s consistency as reflected by numerous fit indices [64]. Hence, the current study employs CMIN, SRMR, CFI, TLI, PGFI, and RMSEA to assess the goodness of fit of a given model to the observed data. Thus, the results suggested a good fit for each dimension’s factors model based on the indices revealed following CFA.

**Fig. 3:**
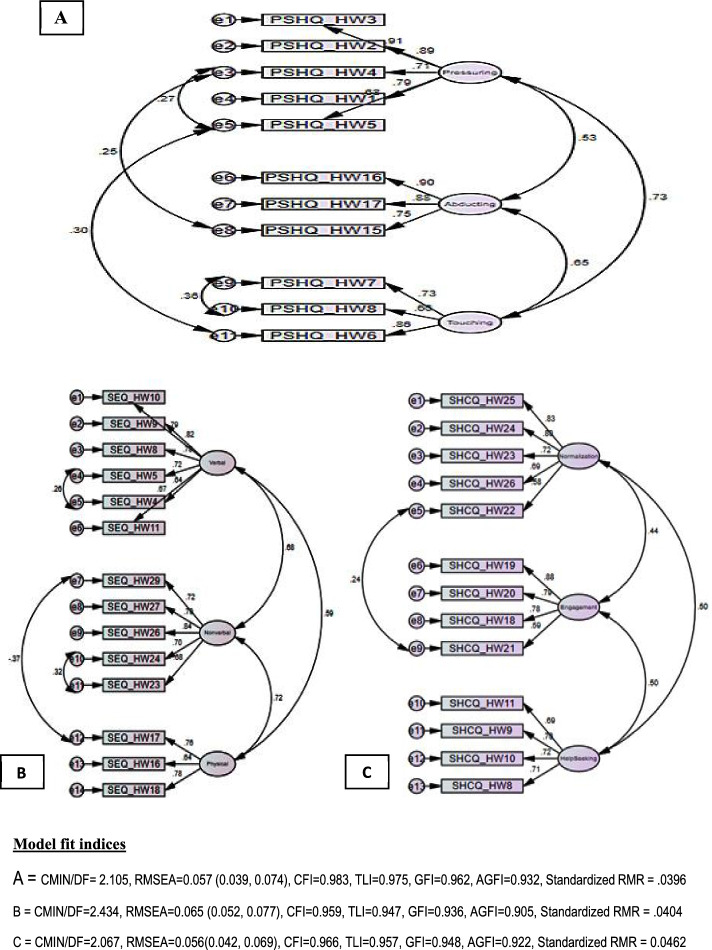
Confirmatory factor analysis of **A**) perceptions of sexual harassment, **B**) Experiences of sexual harassment, and **C**) coping with sexual harassment among women working in Bahir Dar city hospitality workplaces between July 1 and August 30, 2021

Confirmatory factor analysis confirmed the presumed theoretical model ideally since standardized regression coefficients between the factors and the individual items were > 0.51, except (*p*-value < 0.05 in all cases) (Fig. 3). As shown in the figure, the correlations between factors for perception were 0.53, 0.65 and 0.73. Similarly, the correlations between factors for SH experiences were 0.59, 0.68, and 0.72. The correlations between factors for coping with SH techniques were also 0.44, 0.50, and 0.50. The *p*-values for all co-variances between factors were < 0.05. Finally, the adequacy of models that fit the data is entirely acceptable (Fig. 3) [65].

Three factors were identified from each questionnaire to consider the factor analysis results. For SH perception, three factors were identified (Table 1). Similarly, for SH experiences, three factors (i.e., Verbal, non-verbal and physical) were identified, and the items were included in each one (Table 2). Furthermore, three factors (i.e., normalisation, engagement, and help-seeking) were identified, and the items were included in each factor (Table 3).

### Analysis of reliability and validity

Reliability analysis was performed on several indicators for each construct to determine their internal consistency [66]. The dependability of the factors was assessed using the Composite Reliability coefficient. The composite reliability calculates a group of latent construct indicators that assess the same construct [67]. Cronbach’s Alpha has long been used to assess the consistency of a construct. However, it has recently been discovered that relying solely on the Alpha coefficient to determine reliability may be insufficient [68]. Hence, composite reliability is used, which draws on each item’s specified loadings and measurement errors [61]. All composite reliability ratings indicated that the study’s measurements were reliable [69].

Concurrent validity was used to determine the extent to which scale scores have a stronger relationship with criterion measurements made near administration using Pearson product-moment correlation. Taking the sample size (n) of 345, df of 343, and two-tailed (95%CI), the corresponding critical correlation value (r_c_) for a significance level of *α* = 0.05 for a two-tailed test is r_c_ = 0.106*.* Then comparing the composite correlation and each items correlations with the product-moment correlation indicated significance (*P* < 0.001) (SA 6. A, B, and C). For predictive validity, we regressed perceptions, experiences, and coping mechanisms on each subscale. All subscales of perceptions, experiences and coping techniques were significantly associated with their behaviours (SA 7. A & B).

The declaration of convergent validity was based on reliability **(**CR > 0.7), Average variance extracted **(**AVE > 0.5), and discriminant validity **(**MSV < AVE and square root of AVE greater than inter-construct correlations) [60, 61, 70]. As shown in the table, the composite reliability of all factors was greater than 0.7, which is good. The average variance extracted (AVE) for all constructs was greater than 0.5, indicating convergent validity. The square roots of all the AVEs were greater than the correlation between the various constructs, all MSVs were less than the AVE, and all square roots of AVE were greater than inter-construct correlations. Therefore, there was discriminant validity (Table 4). Furthermore, all dimensions of SH perceptions, experiences, and coping techniques had a moderate to high correlation.

**Table 4:** Tests of convergent and discriminant validity for sexual harassment perceptions, experiences, and coping techniques among women working in Bahir Dar city hospitality workplaces between July 1 and August 30, 2021

Perceptions	CR	AVE	MSV	MaxR(H)	Abducting	Pressuring	Touching
**Abducting**	0.883	0.716	0.425	0.901	**0.846**		
**Pressuring**	0.898	0.641	0.529	0.955	0.527	**0.800**	
**Touching**	0.799	0.573	0.529	0.963	0.652	0.727	**0.757**
**Experiences**	CR	AVE	MSV	MaxR(H)	**Nonverbal**	**Verbal**	**Physical**
**Nonverbal**	0.866	0.565	0.518	0.877	**0.752**		
**Verbal**	0.877	0.546	0.468	0.937	0.684	**0.739**	
**Physical**	0.772	0.532	0.518	0.949	0.720	0.587	**0.729**
**Coping**	CR	AVE	MSV	MaxR(H)	**Engagement**	**Normalization**	**Help Seeking**
**Engagement**	0.847	0.585	0.246	0.876	**0.765**		
**Normalization**	0.848	0.531	0.253	0.931	0.439	**0.729**	
**Help Seeking**	0.813	0.521	0.253	0.947	0.496	0.503	**0.722**

## Discussion

This study aimed to develop a culturally appropriate measure of SH perceptions, experiences and coping techniques in the hospitality workplaces in Ethiopia, which would serve as a platform for other researchers, non-governmental organisations, and government officials involved in eliminating SH and its implications for women working in the hospitality workplaces. This research is an essential part of evaluating SH in the hospitality industry and is a helpful instrument for assessing the various dimensions of SH perceptions, experiences and coping. It thus provided helpful information for developing appropriate reproductive health care management methods in hospitality workplaces. Policies and programs can also be established following enhanced metrics and a greater understanding of the issue’s prevalence.

The approach suggested by Boateng et al. [45] for health, social and behavioural research in the development and validation process was used. Content validity was done after item generation and refining, where the meaning for establishing an instrument’s validity is debatable [71]. On the other hand, the two-stage approach is widely accepted in the methodological literature and is required for developing new instruments [72, 73]. The critical point of contention is calculating the agreement indices and the number of experts in determining the risk of inaccuracy. The modified kappa was employed as an index to account for chance agreement, and this index provides data on the degree of agreement that is not determined by chance. Questions about whether alternate methods, such as multi-rater kappa, would produce a correct agreement cannot be answered here.

Moreover, the agreement indices are simply one component in determining content validity and must not be used as the only basis for rejecting or modifying items [74]. The validity of the SH perceptions, experiences and coping techniques scales was supported in this study by the development process of the instrument and the evaluation of its content validity. Experts’ opinions were aided in making decisions regarding the types of issues that existed for specific items. Thus, combined with adjusted kappa and expert-written comments, CVI brought rigour to the content validation of SH perceptions, experiences, and coping techniques measures. This implies that we used the representation of currently available knowledge in the construct of interest, content validity [28]. Content validity, along with face validity, is the minimum quality criteria. Despite its low place in the validity hierarchy [14, 74], content validity is a significant quality indicator of an instrument validity and provides insight into its feasibility and practicability [72, 75, 76].

In other situations, the written remarks also revealed problems with comprehension, indicating that the intended message was misconstrued. Because of this information, the item was rephrased to be more precise. The importance of the expert’s competence cannot be overstated. Various characteristics can be utilised to certify a person’s status as an expert, and there are no fixed standards for defining an expert. An expert is characterised as representing a topic of interest in research practice. Knowledge of the assessment technique is fundamental in instrument assessment, and input from stakeholders from other application sectors might be useful [75]. We focused on experiences, perceptions, and coping mechanisms of SH among women, in hospitality work in the context of hospitality industries and using or developing assessment instruments in the first expert panel of this study. We requested a strong focus on women working in the hospitality industry, as we expected those experts to contribute to developing an instrument that is meaningful, understandable, and practical for women working in the industry. The level to which the narrow focus on hospitality workplaces influenced the relevance of the SH perceptions, experiences, and coping techniques scales and dimensions could not be determined. Because most experts had additional qualifications, we assume bias is a small risk.

This second modification heavily debated problems acquiring the information needed to answer the questions. Women working in the hospitality industry were frequently reliant on information provided by senior employees or managers if they were there. However, in some situations, these informants were unavailable, and the information needed to answer the questions about SH perceptions, experiences and coping techniques was complex. This is not specific to SH perceptions, experiences, and coping techniques but a general problem of assessing complex and multifaceted information, creating a professional environment where the observation, collection, communication, reflection, and appraisal of essential information is a prominent part of the hospitality workplace process, overcoming this challenge. Creating a professional environment where the customers are respected is a prominent part of the hospitality industry.

The content was vague. The relevance of the SH perceptions, experiences and coping techniques information seemed unclear to most respondents. This finding corroborated the notion that SH perceptions, experiences, and coping mechanisms necessitated a broad understanding. This feedback was required to incorporate the SH perceptions, experiences and coping techniques in the future. The importance of describing the links between the variable of SH perspectives, experiences, and coping mechanisms, and their significance to the research of SH related difficulties, should be emphasised. However, the context in which women encounter SH impacts their understanding and coping with it. The influence of cognition on the meaning formation of SH behaviours is powerful. Thus, women employees in the hospitality workplace must adopt more person-centred attitudes towards perpetrators.

Pearson’s correlation coefficient created the convergent validity of the SH perceptions, experiences, and coping technique factors. The correlations were positive, even high in certain situations, indicating similar constructs. The correlations’ results proved this validity since they met the criteria provided by Devon et al. [76] for this type of validity. Composite reliability was used to establish the instrument’s convergent validity in the second test. Test values of > 0.6 was considered acceptable [61, 76–78]. Both tests revealed that this form of validity exists. The contrasts between the several elements that made up the scale reflected its discriminant validity. This form of validity emerges when the concepts that make up the system are distinct and related. To establish the validity of these principles, they were evaluated in various methods. The first evaluation involved comparing the AVE’s square root to the correlation between the scale’s constructs [61]. To ensure discriminant validity, the square root of the AVE should be greater than the correlation between the constructs. By looking at the correlations and AVE values, it can be concluded that they have discriminant validity.

There are two different techniques to confirm the validity of this form of validity. Burnkrant and Page [78] have proposed the first. It tries to estimate alternative models so that they all have the same restriction, namely that the correlation between each pair of dimensions must be equal to 1. On the other hand, each model should be compared using a chi-square test to see if they are statistically different. According to our findings, the differences in the chi-squared values were usually significant. Therefore, the dimensions of SH perceptions, experiences, and coping techniques were distinct, indicating discriminant validity. The third method is computing the possible correlations between the factors and constructing the confidence intervals for all dimension’s correlations. The current study’s findings also confirm the presence of this form of validity, as none of the correlations’ confidence intervals had the value one at 95% confidence [79].

The scales for SH perceptions, experiences and coping techniques can be seen as tools that can help women and supervisors better understand SH. This program provides instructions for traversing a complex web of potential causes and triggers for certain behaviours. In comparison to other questionnaires used in previous surveys in hospitality workplaces, this tool focused on measuring employees’ perceptions and coping mechanisms in addition to evaluating experiences. It is essential to note that the SH perceptions, experiences and coping techniques do not provide specific solutions to the problem; instead, they aid in generating a hypothesis (or more) concerning SH perceptions, experiences, and coping.

The estimation and consideration of these scores did not only help select the items for the final survey, but it also allowed prioritizing which opportunities for improvement were most important from the employee’s perspective during work and implementation. Based on the employees’ experiences, along with perceptions and coping tools, the improvement of the hospitality workplaces women employee’s safety may be established within the framework of continuously improving its quality, maximizing the acceptability of the hospitality services by their users, and strengthening the accessibility to services and equity. This knowledge is essential for managers and women employees in this sector. It will provide them with valuable insight into the quality of the provided services and help them proceed to meaningful comparisons amongst their counterparts to identify best practices and priority areas for potential improvement. Therefore, we recommended that these evaluation surveys be used as an ongoing evaluation tool in everyday hospitality workplace SH management practices.

This measurement development and validation process might also help design prevalence studies that use validated measures for SH in hospitality workplaces of low- and middle-income counties, which will enable us to obtain more precise prevalence estimates across hospitality workplaces to design effective interventions and policies [26]. It might also further update the SH questionnaires utilised in different settings [80].

## Conclusions

The assessment methods that focus on women working in the hospitality industry are SH perceptions, experiences, and coping techniques. The SH perceptions, experiences, and coping scales provide a locally verified method to comprehensively assess SH in Ethiopia by government authorities and local and international non-governmental organisations, which in turn will aid in providing necessary services and the evaluation of efforts to improve workplace safety health psychosocial well-being. The content validity of the SH perceptions, experiences, and coping technique questionnaires is enhanced by the qualitative study findings, comprehensive literature review, solid theoretical foundation, and the two evaluations representing specific content expertise and practical perspectives. As a result, these help in measuring perceptions, experiences, and coping mechanisms of sexual harassment in low and middle-income countries, which aid in providing necessary services and the evaluation of efforts to improve workplace safety, health, and psychosocial well-being. However, because the SH perceptions, experiences, and coping technique requires observation, it is recommended to be used as a research tool in Ethiopian hospitality workplaces and other low and middle-income countries. Thus, future studies would ensure that the instrument is checked for improved applicability in diverse work settings.

## Supplementary Information

**Additional file 1: SA 1.** The identified domains, definitions, and dimensions in developing and validating sexual harassment perception, experiences and coping mechanisms questionnaires for women working in Bahir Dar city hospitality workplaces between July 1 and August 30, 2021. **SA 2.** The content validity assessments**. SA 3.** Cognitive Protocol. **SA 4.** Survey questionnaires. **SA 5.** Participants’ characteristics and working organisation related to Bahir Dar city hospitality workplaces between July 1 and August 30, 2021. **SA 6.** Pearsons’s product-moment correlations for concurrent validity. **SA 7.** Predictive Validity.

## Data Availability

The datasets used and/or analysed during the current study are available from the corresponding author on reasonable request.

## Abbreviations

AMOS: Analysis of Moment Structure
ASV: Average shared variance
AVE: Average variance extracted
CFA: Confirmatory factor Analysis
CFI: Confirmatory Fit Index
CR: composite reliability
EFI: Exploratory Factor Analysis
HIV: Human immunodeficiency Virus
IFI: Incremental Fit Index
IRB: Institutional Review Board
KMO: Kaiser-Meyer-Olkien
MaxR(H): Maximum reliability
MPH: Master of Public Health
MSc: Master of Science
MSV: Maximum shared variance
NGOs: Non-governmental Organisations
NY: New York
PhD: Doctor of Philosophy
PSHQ_HW: perception sexual harassment questionnaire for hospitality workplaces
RMSEA: root mean square error of approximation
SA: Supplementary Appendix
SD: Standard deviation
SEQ_HW: sexual harassment experiences questionnaire for hospitality workplaces
SH: Sexual Harassment
SHCQ_HW: sexual harassment coping mechanisms for hospitality workplaces
SPSS: Statistical Package for social sciences
SRMR: standardised root mean square residual
STIs: Sexually transmitted infections
USA: United States of America
