# Supplementary material for: Development and validation of contextual measures of sexual harassment perceptions, experiences, and coping for women employees in Ethiopian hospitality workplaces
Source: Arch Public Health. 2022 Feb 18;80:59. doi: 10.1186/s13690-022-00828-z (PMC8857844; doi:10.1186/s13690-022-00828-z)
Supplement: Supplementary file 1 — Additional file 1: SA 1. The identified domains, definitions, and dimensions in developing and validating sexual harassment perception, experiences and coping mechanisms questionnaires for women working in Bahir Dar city hospitality workplaces between July 1 and August 30, 2021. SA 2. The content validity assessments. SA 3. Cognitive Protocol. SA 4. Survey questionnaires. SA 5. Participants’ characteristics and working organisation related to Bahir Dar city hospitality workplaces between July 1 and August 30, 2021. SA 6. Pearsons’s product-moment correlations for concurrent validity. SA 7. Predictive Validity. [file 13690_2022_828_MOESM1_ESM.docx]

**SA 1.** **The identified domains, their definitions and dimensions in the development and validation of tools for women working in hospitality workplaces**

| **Domains** | **Definitions** | **Dimensions** |
| --- | --- | --- |
| SH Perceptions | Any conduct perceived as unwelcome or undesired that women hospitality workplace employees view as offensive creates an intimidating or offensive work environment that interferes with women’s ability to do their job. | Pressuring, Threatening, Touching, Forcing |
| SH experiences | An encounter of women is delineated to be sexually unwanted, coercive, and undermine gender issues. | verbal, non-verbal, physical |
| SH coping | Coping depicts how people detect, appraise, deal with, and learn from stressful encounters. | Normalisation, Engagement, Help-seeking, Detachment |

**SA 2. The content validity assessments**

1. **Perception**

| Item contents | | Relevance of the questionnaire | | | | | | | Necessity of the questionnaire | | | | | | | Clarity of the questionnaire | | | | | |
| --- | --- | --- | --- | --- | --- | --- | --- | --- | --- | --- | --- | --- | --- | --- | --- | --- | --- | --- | --- | --- | --- |
| Item code | Item Name | | No of experts | No of ratings of 3 or 4 | I-CVI | Pc | cK* | Evaluation | ratings of 4 or 5 | I-CVI | I-CVR | Pc | cK* | Evaluation | ratings of 4 or 5 | | I-CVI | I-CVR | Pc | cK* | Evaluation |
| Sexual harassment is… | | |  |  |  |  |  |  |  |  |  |  |  |  |  |  |  |  |  |  |  |
| PSHQ1 | …preparing women for sexual harassment by giving them exaggerated bonuses | | 5 | 4 | 0.8 | 0.16 | 0.54 |  | 5 | 1 | 1 | 0.03 | 0.94 |  | 5 | | 1 | 1 | 0.03 | 0.94 |  |
| PSHQ2 | …preparing for sexual harassment by making inappropriate promises | | 5 | 5 | 1 | 0.03 | 0.94 |  | 5 | 1 | 1 | 0.03 | 0.94 |  | 5 | | 1 | 1 | 0.03 | 0.94 |  |
| PSHQ3 | … preparing women for sexual harassment by providing better career advancement | | 5 | 5 | 1 | 0.03 | 0.94 |  | 5 | 1 | 1 | 0.03 | 0.94 |  | 5 | | 1 | 1 | 0.03 | 0.94 |  |
| PSHQ4 | … an offer of a new job in exchange for sexual advances | | 5 | 5 | 1 | 0.03 | 0.94 |  | 5 | 1 | 1 | 0.03 | 0.94 |  | 5 | | 1 | 1 | 0.03 | 0.94 |  |
| PSHQ5 | …an act of threatening to hurt relatives in exchange for sexual advances | | 5 | 3 | 0.6 | 0.04 | 0.54 |  | 3 | 0.6 | 0.2 | 0.04 | 0.54 |  | 3 | | 0.6 | 0.2 | 0.04 | 0.15 |  |
| PSHQ6 | … an act of intimidation to fire a woman unless she agrees to have sex | | 5 | 4 | 0.8 | 0.16 | 0.54 |  | 4 | 0.8 | 0.6 | 0.16 | 0.54 |  | 5 | | 1 | 1 | 0.03 | 0.94 |  |
| PSHQ7 | … threatening to complain or falsely accuse about the service being provided to the immediate supervisor unless women agree to sexual favours | | 5 | 5 | 1 | 0.03 | 0.94 |  | 4 | 0.8 | 0.6 | 0.16 | 0.54 |  | 5 | | 1 | 1 | 0.03 | 0.94 |  |
| PSHQ8 | … an act of touching sexual sensitive parts while the women are at work | | 5 | 5 | 1 | 0.03 | 0.94 |  | 4 | 0.8 | 0.6 | 0.16 | 0.54 |  | 5 | | 1 | 1 | 0.03 | 0.94 |  |
| PSHQ9 | … an act of speaking random sexual jokes to women while she is at work | | 5 | 5 | 1 | 0.03 | 0.94 |  | 5 | 1 | 1 | 0.03 | 0.94 |  | 5 | | 1 | 1 | 0.03 | 0.94 |  |
| PSHQ10 | … a repeated request of women to engage in sexual activities | | 5 | 5 | 1 | 0.03 | 0.94 |  | 5 | 1 | 1 | 0.03 | 0.94 |  | 5 | | 1 | 1 | 0.03 | 0.94 |  |
| PSHQ11 | … an act of emotionally distressing women based on their gender | | 5 | 5 | 1 | 0.03 | 0.94 |  | 3 | 0.6 | 0.2 | 0.04 | 0.54 |  | 4 | | 0.8 | 0.6 | 0.16 | 0.37 |  |
| PSHQ12 | … an act of poking women with a finger, foot, or pointed object while they are at work | | 5 | 5 | 1 | 0.03 | 0.94 |  | 4 | 0.8 | 0.6 | 0.16 | 0.54 |  | 5 | | 1 | 1 | 0.03 | 0.94 |  |
| PSHQ13 | … a request telephone number of women while they are at work | | 5 | 5 | 1 | 0.03 | 0.94 |  | 5 | 1 | 1 | 0.03 | 0.94 |  | 5 | | 1 | 1 | 0.03 | 0.94 |  |
| PSHQ14 | … an act of showing pornographic movies/pictures to women while they are at work | | 5 | 5 | 1 | 0.03 | 0.94 |  | 5 | 1 | 1 | 0.03 | 0.94 |  | 5 | | 1 | 1 | 0.03 | 0.94 |  |
| PSHQ15 | … writing sexual messages on the pay bill to women while they are at work | | 5 | 5 | 1 | 0.03 | 0.94 |  | 3 | 0.6 | 0.2 | 0.04 | 0.54 |  | 4 | | 0.8 | 0.6 | 0.16 | 0.37 |  |
| PSHQ16 | … an unfair treatment of women because of their gender | | 5 | 5 | 1 | 0.03 | 0.94 |  | 3 | 0.6 | 0.2 | 0.04 | 0.54 |  | 5 | | 1 | 1 | 0.03 | 0.94 |  |
| PSHQ17 | … an act of undermining women because of their gender | | 5 | 4 | 0.8 | 0.16 | 0.54 |  | 2 | 0.4 | -0.2 | 0.35 | 0.03 |  | 4 | | 0.8 | 0.6 | 0.16 | 0.37 |  |
| PSHQ18 | … a forcible take of woman for sexual intercourse after they leave their job | | 5 | 5 | 1 | 0.03 | 0.94 |  | 2 | 0.4 | -0.2 | 0.35 | 0.03 |  | 3 | | 0.6 | 0.2 | 0.04 | 0.15 |  |
| PSHQ19 | … conducting sexual intercourse through force or under threat of injury against the women will | | 5 | 5 | 1 | 0.03 | 0.94 |  | 5 | 1 | 1 | 0.03 | 0.94 |  | 5 | | 1 | 1 | 0.03 | 0.94 |  |
| PSHQ20 | … slapping, kicking, pinching, or insulting women while they refuse to agree to a sexual favour | | 5 | 5 | 1 | 0.03 | 0.94 |  | 5 | 1 | 1 | 0.03 | 0.94 |  | 5 | | 1 | 1 | 0.03 | 0.94 |  |
|  | | | | S-CVI/Ave | 0.95 | cK*/Ave=0.86 | | | | S-CVI/Ave | 0.82 | Kappa/Ave=0.69 | | | | | S-CVI/Ave | 0.93 | S-CVR/Ave | 0.86 |  |
|  |  |  |  | Total Agreement | 16 |  |  |  |  | Total Agreement | 14 |  |  |  |  |  | Total Agreement | 18 | cK*/Ave = 0.78 | | |
|  |  |  |  | S-CVI/UA | 0.8 |  |  |  |  | S-CVI/UA | 0.7 |  |  |  |  |  | S-CVI/UA | 0.9 |  |  |  |
|  |  |  |  | Sum-CVI | 19 |  |  |  |  | Sum-CVI | 16.4 |  |  |  |  |  | Sum-CVI | 18.6 |  |  |  |
|  |  |  |  | CVR | 0.95 |  |  |  |  | CVR | 0.82 |  |  |  |  |  | CVR | 0.93 |  |  |  |

1. Expereinces

| Item contents | | Relevance of the questionnaire | | | | | | Necessity of the questionnaire | | | | | | Clarity of the questionnaire | | | | | |
| --- | --- | --- | --- | --- | --- | --- | --- | --- | --- | --- | --- | --- | --- | --- | --- | --- | --- | --- | --- |
| Item code | Item Name | No of experts | No of ratings of 3 or 4 | I-CVI | Pc | cK* | Evaluation | No of ratings of 4 or 5 | I-CVI | I-CVR | Pc | cK* | Evaluation | No of ratings of 4 or 5 | I-CVI | I-CVR | Pc | cK* | Evaluation |
| How often have you tried to… | |  |  |  |  |  |  |  |  |  |  |  |  |  |  |  |  |  |  |
| SHCQ1 | …file a formal complaint about sexual harassment? | 5 | 5 | 1 | 0.03 | 0.94 |  | 5 | 1 | 1 | 0.03 | 0.94 |  | 5 | 1 | 1 | 0.03 | 0.94 |  |
| SHCQ2 | …report the perpetrator of sexual harassment? | 5 | 5 | 1 | 0.03 | 0.94 |  | 5 | 1 | 1 | 0.03 | 0.94 |  | 5 | 1 | 1 | 0.03 | 0.94 |  |
| SHCQ3 | …talk with supervisors, managers, and unions about the event of SH? | 5 | 5 | 1 | 0.03 | 0.94 |  | 5 | 1 | 1 | 0.03 | 0.94 |  | 5 | 1 | 1 | 0.03 | 0.94 |  |
| SHCQ4 | …tell the perpetrator that you do not like sexual harassment? | 5 | 4 | 0.8 | 0.16 | 0.54 |  | 2 | 0.4 | -0.2 | 0.35 | 0.03 |  | 5 | 1 | 1 | 0.03 | 0.94 |  |
| SHCQ5 | …ask the perpetrator to leave you alone? | 5 | 4 | 0.8 | 0.16 | 0.54 |  | 2 | 0.4 | -0.2 | 0.35 | 0.03 |  | 5 | 1 | 1 | 0.03 | 0.94 |  |
| SHCQ6 | …forget about the event of sexual harassment? | 5 | 5 | 1 | 0.03 | 0.94 |  | 5 | 1 | 1 | 0.03 | 0.94 |  | 5 | 1 | 1 | 0.03 | 0.94 |  |
| SHCQ7 | …tell yourself that it was not necessary to think about SH? | 5 | 4 | 0.8 | 0.16 | 0.54 |  | 5 | 1 | 1 | 0.03 | 0.94 |  | 5 | 1 | 1 | 0.03 | 0.94 |  |
| SHCQ8 | …play games or engage in outdoor activities to forget about SH event? | 5 | 5 | 1 | 0.03 | 0.94 |  | 3 | 0.6 | 0.2 | 0.04 | 0.54 |  | 3 | 0.6 | 0.2 | 0.04 | 0.54 |  |
| SHCQ9 | …watching TV more than usual to forget about the event of SH? | 5 | 5 | 1 | 0.03 | 0.94 |  | 5 | 1 | 1 | 0.03 | 0.94 |  | 5 | 1 | 1 | 0.03 | 0.94 |  |
| SHCQ10 | …going to a friend for advice on how to change the situation? | 5 | 5 | 1 | 0.03 | 0.94 |  | 5 | 1 | 1 | 0.03 | 0.94 |  | 5 | 1 | 1 | 0.03 | 0.94 |  |
| SHCQ11 | …get sympathy and understanding from friends who have had the same problem? | 5 | 5 | 1 | 0.03 | 0.94 |  | 5 | 1 | 1 | 0.03 | 0.94 |  | 5 | 1 | 1 | 0.03 | 0.94 |  |
| SHCQ12 | …talk to people about the situation because talking about it makes you feel better? | 5 | 5 | 1 | 0.03 | 0.94 |  | 5 | 1 | 1 | 0.03 | 0.94 |  | 2 | 0.4 | -0.2 | 0.35 | 0.03 |  |
| SHCQ13 | …seek reassurance from those who know you best? | 5 | 5 | 1 | 0.03 | 0.94 |  | 4 | 0.8 | 0.6 | 0.16 | 0.54 |  | 4 | 0.8 | 0.6 | 0.16 | 0.54 |  |
| SHCQ14 | …share your fears and anxieties with a friend or relative? | 5 | 4 | 0.8 | 0.16 | 0.54 |  | 3 | 0.6 | 0.2 | 0.04 | 0.54 |  | 4 | 0.8 | 0.6 | 0.16 | 0.54 |  |
| SHCQ15 | …set some goals for yourself to deal with the situation? | 5 | 5 | 1 | 0.03 | 0.94 |  | 3 | 0.6 | 0.2 | 0.04 | 0.54 |  | 4 | 0.8 | 0.6 | 0.16 | 0.54 |  |
| SHCQ16 | …thought about all the solutions before deciding what to do? | 5 | 4 | 0.8 | 0.16 | 0.54 |  | 4 | 0.8 | 0.6 | 0.16 | 0.54 |  | 4 | 0.8 | 0.6 | 0.16 | 0.54 |  |
| SHCQ17 | …carefully plan a course of action rather than acting on impulse? | 5 | 5 | 1 | 0.03 | 0.94 |  | 5 | 1 | 1 | 0.03 | 0.94 |  | 4 | 0.8 | 0.6 | 0.16 | 0.54 |  |
| SHCQ18 | …decided not to participate in hospitality work? | 5 | 5 | 1 | 0.03 | 0.94 |  | 3 | 0.6 | 0.2 | 0.04 | 0.54 |  | 5 | 1 | 1 | 0.03 | 0.94 |  |
| SHCQ19 | …pray while experiencing sexual harassment? | 5 | 5 | 1 | 0.03 | 0.94 |  | 4 | 0.8 | 0.6 | 0.16 | 0.54 |  | 3 | 0.6 | 0.2 | 0.04 | 0.54 |  |
| SHCQ20 | …changed your job frequently because of sexual harassment? | 5 | 5 | 1 | 0.03 | 0.94 |  | 5 | 1 | 1 | 0.03 | 0.94 |  | 5 | 1 | 1 | 0.03 | 0.94 |  |
| SHCQ21 | …bring the perpetrator to justice? | 5 | 5 | 1 | 0.03 | 0.94 |  | 3 | 0.6 | 0.2 | 0.04 | 0.54 |  | 5 | 1 | 1 | 0.03 | 0.94 |  |
| SHCQ22 | …consult a health care provider because of sexual harassment? | 5 | 5 | 1 | 0.03 | 0.94 |  | 4 | 0.8 | 0.6 | 0.16 | 0.54 |  | 5 | 1 | 1 | 0.03 | 0.94 |  |
| SHCQ23 | …consult a psychologist because of the SH that you face? | 5 | 5 | 1 | 0.03 | 0.94 |  | 3 | 0.6 | 0.2 | 0.04 | 0.54 |  | 5 | 1 | 1 | 0.03 | 0.94 |  |
| SHCQ24 | … negotiate with the perpetrator? | 5 | 5 | 1 | 0.03 | 0.94 |  | 5 | 1 | 1 | 0.03 | 0.94 |  | 5 | 1 | 1 | 0.03 | 0.94 |  |
| SHCQ25 | …threaten the perpetrator? | 5 | 3 | 0.6 | 0.04 | 0.54 |  | 3 | 0.6 | 0.2 | 0.04 | 0.54 |  | 4 | 0.8 | 0.6 | 0.16 | 0.54 |  |
| SHCQ26 | …discriminate against the perpetrators? | 5 | 5 | 1 | 0.03 | 0.94 |  | 4 | 0.8 | 0.6 | 0.16 | 0.54 |  | 4 | 0.8 | 0.6 | 0.16 | 0.54 |  |
| SHCQ27 | …reject the request for sexual harassment? | 5 | 5 | 1 | 0.03 | 0.94 |  | 4 | 0.8 | 0.6 | 0.16 | 0.54 |  | 5 | 1 | 1 | 0.03 | 0.94 |  |
| SHCQ28 | …confront the perpetrator? | 5 | 5 | 1 | 0.03 | 0.94 |  | 5 | 1 | 1 | 0.03 | 0.94 |  | 5 | 1 | 1 | 0.03 | 0.94 |  |
| SHCQ29 | …tolerate sexual harassment? | 5 | 5 | 1 | 0.03 | 0.94 |  | 4 | 0.8 | 0.6 | 0.16 | 0.54 |  | 4 | 0.8 | 0.6 | 0.16 | 0.54 |  |
| SHCQ30 | …keep silent to respond to sexual harassment? | 5 | 5 | 1 | 0.03 | 0.94 |  | 5 | 1 | 1 | 0.03 | 0.94 |  | 5 | 1 | 1 | 0.03 | 0.94 |  |
| SHCQ31 | …ignore sexual harassment? | 5 | 5 | 1 | 0.03 | 0.94 |  | 4 | 0.8 | 0.6 | 0.16 | 0.54 |  | 3 | 0.6 | 0.2 | 0.04 | 0.54 |  |
| SHCQ32 | …accept sexual harassment? | 5 | 5 | 1 | 0.03 | 0.94 |  | 4 | 0.8 | 0.6 | 0.16 | 0.54 |  | 5 | 1 | 1 | 0.03 | 0.94 |  |
|  | | | S-CVI/Ave | 0.96 | cK*/Ave | 0.86 |  | S-CVI/Ave | 0.82 |  |  |  |  | S-CVI/Ave | 0.89 |  | cK*/Ave | 0.77 |  |
|  |  |  | Total Agree | 26 |  | | | Agreement | 23 | s-CVR | 0.82 |  |  | Agreement | 20 |  |  | | |
|  |  |  | S-CVI/UA | 0.81 |  |  |  | S-CVI/UA | 0.72 |  | cK*/Ave | 0.68 |  | S-CVI/UA | 0.63 |  |  |  |  |
|  |  |  | Sum-CVI | 30.6 |  |  |  | Sum-CVI | 26.2 |  |  |  |  | Sum-CVI | 28.6 |  |  |  |  |
|  |  |  | CVR | 0.96 |  |  |  |  |  |  |  |  |  | S-CVR/Ave | 0.79 |  |  |  |  |

1. Copings

| Item contents | | Relevance of the questionnaire | | | | | | Necessity of the questionnaire | | | | | | Clarity of the questionnaire | | | | | |
| --- | --- | --- | --- | --- | --- | --- | --- | --- | --- | --- | --- | --- | --- | --- | --- | --- | --- | --- | --- |
| Item code | Item Name | No of experts | No of ratings of 3 or 4 | I-CVI | Pc | cK* | Evaluation | No of ratings of 4 or 5 | I-CVI | I-CVR | Pc | cK* | Evaluation | No of ratings of 4 or 5 | I-CVI | I-CVR | Pc | cK* | Evaluation |
| How often have you tried to… | |  |  |  |  |  |  |  |  |  |  |  |  |  |  |  |  |  |  |
| SEQ1 | …offered you rewards in return for your cooperation in sexual matters? | 5 | 5 | 1 | 0.03 | 0.94 |  | 5 | 1 | 1 | 0.03 | 0.94 |  | 5 | 1 | 1 | 0.03 | 0.94 |  |
| SEQ2 | … mistreat you for your refusal of cooperating with sexual matters? | 5 | 5 | 1 | 0.03 | 0.94 |  | 4 | 0.8 | 0.6 | 0.16 | 0.54 |  | 5 | 1 | 1 | 0.03 | 0.94 |  |
| SEQ3 | …tell you sexually explicit jokes? | 5 | 5 | 1 | 0.03 | 0.94 |  | 5 | 1 | 1 | 0.03 | 0.94 |  | 5 | 1 | 1 | 0.03 | 0.94 |  |
| SEQ4 | … sexually assaulted you in public or in private? | 5 | 5 | 1 | 0.03 | 0.94 |  | 4 | 0.8 | 0.6 | 0.16 | 0.54 |  | 5 | 1 | 1 | 0.03 | 0.94 |  |
| SEQ5 | …have unwanted sexual conversations with you? | 5 | 5 | 1 | 0.03 | 0.94 |  | 3 | 0.6 | 0.2 | 0.04 | 0.54 |  | 3 | 0.6 | 0.2 | 0.04 | 0.54 |  |
| SEQ6 | …did the perpetrator repeatedly ask you for sexual dates even after you refused? | 5 | 5 | 1 | 0.03 | 0.94 |  | 5 | 1 | 1 | 0.03 | 0.94 |  | 4 | 0.8 | 0.6 | 0.16 | 0.54 |  |
| SEQ7 | …attempts to establish a sexual relationship with you? | 5 | 5 | 1 | 0.03 | 0.94 |  | 5 | 1 | 1 | 0.03 | 0.94 |  | 3 | 0.6 | 0.2 | 0.04 | 0.54 |  |
| SEQ8 | …touch you in a way that made you feel uncomfortable? | 5 | 5 | 1 | 0.03 | 0.94 |  | 5 | 1 | 1 | 0.03 | 0.94 |  | 5 | 1 | 1 | 0.03 | 0.94 |  |
| SEQ9 | … handle you differently because of your sex? | 5 | 3 | 0.6 | 0.04 | 0.54 |  | 3 | 0.6 | 0.2 | 0.04 | 0.54 |  | 2 | 0.4 | -0.2 | 0.35 | 0.03 |  |
| SEQ10 | …made unnecessary contact with you, or have violated your personal boundaries? | 5 | 5 | 1 | 0.03 | 0.94 |  | 3 | 0.6 | 0.2 | 0.04 | 0.54 |  | 3 | 0.6 | 0.2 | 0.04 | 0.54 |  |
| SEQ11 | …hug you in a way that made you feel uncomfortable? | 5 | 4 | 0.8 | 0.16 | 0.54 |  | 4 | 0.8 | 0.6 | 0.16 | 0.54 |  | 3 | 0.6 | 0.2 | 0.04 | 0.54 |  |
| SEQ12 | …target you for rumours of sexual promiscuity? | 5 | 5 | 1 | 0.03 | 0.94 |  | 4 | 0.8 | 0.6 | 0.16 | 0.54 |  | 5 | 1 | 1 | 0.03 | 0.94 |  |
| SEQ13 | …insult you by targeting your sexual orientation? | 5 | 5 | 1 | 0.03 | 0.94 |  | 5 | 1 | 1 | 0.03 | 0.94 |  | 5 | 1 | 1 | 0.03 | 0.94 |  |
| SEQ14 | … make unwanted comments about your body, clothing, or way of living? | 5 | 5 | 1 | 0.03 | 0.94 |  | 4 | 0.8 | 0.6 | 0.16 | 0.54 |  | 5 | 1 | 1 | 0.03 | 0.94 |  |
| SEQ15 | … put you in a corner where it was difficult to get out? | 5 | 4 | 0.8 | 0.16 | 0.54 |  | 3 | 0.6 | 0.2 | 0.04 | 0.54 |  | 3 | 0.6 | 0.2 | 0.04 | 0.54 |  |
| SEQ16 | … show you sexually explicit images or objects that he found unwanted or unpleasant? | 5 | 5 | 1 | 0.03 | 0.94 |  | 5 | 1 | 1 | 0.03 | 0.94 |  | 4 | 0.8 | 0.6 | 0.16 | 0.54 |  |
| SEQ17 | …make telephone calls to you or gave you letters with sexual content? | 5 | 5 | 1 | 0.03 | 0.94 |  | 5 | 1 | 1 | 0.03 | 0.94 |  | 5 | 1 | 1 | 0.03 | 0.94 |  |
| SEQ18 | …make unwanted inquiries of sexual services with the promise of rewards to you? | 5 | 4 | 0.8 | 0.16 | 0.54 |  | 3 | 0.6 | 0.2 | 0.04 | 0.54 |  | 4 | 0.8 | 0.6 | 0.04 | 0.73 |  |
| SEQ19 | …make unwanted inquiries of sexual services with threats of punishment or sanctions? | 5 | 5 | 1 | 0.03 | 0.94 |  | 3 | 0.6 | 0.2 | 0.04 | 0.54 |  | 4 | 0.8 | 0.6 | 0.04 | 0.73 |  |
| SEQ20 | … make sexual assaults, attempts of rape, or actual rape? | 5 | 5 | 1 | 0.03 | 0.94 |  | 4 | 0.8 | 0.6 | 0.16 | 0.54 |  | 3 | 0.6 | 0.2 | 0.04 | 0.54 |  |
| SEQ21 | …unnecessarily expose themselves in front of you? | 5 | 5 | 1 | 0.03 | 0.94 |  | 5 | 1 | 1 | 0.03 | 0.94 |  | 4 | 0.8 | 0.6 | 0.16 | 0.54 |  |
| SEQ22 | …threaten you by file a complaint about your service to your supervisor because you refused a sexual request? | 5 | 5 | 1 | 0.03 | 0.94 |  | 5 | 1 | 1 | 0.03 | 0.94 |  | 5 | 1 | 1 | 0.03 | 0.94 |  |
| SEQ23 | …affect you by whistling, calling, or sexually honked at you? | 5 | 5 | 1 | 0.03 | 0.94 |  | 3 | 0.6 | 0.2 | 0.04 | 0.54 |  | 4 | 0.8 | 0.6 | 0.16 | 0.54 |  |
| SEQ24 | …make unwelcome attempts to draw you into a discussion of sexual matters? | 5 | 5 | 1 | 0.03 | 0.94 |  | 4 | 0.8 | 0.6 | 0.16 | 0.54 |  | 5 | 1 | 1 | 0.03 | 0.94 |  |
| SEQ25 | …make offensive remarks about your appearance, body, or sexual activities? | 5 | 5 | 1 | 0.03 | 0.94 |  | 4 | 0.8 | 0.6 | 0.16 | 0.54 |  | 4 | 0.8 | 0.6 | 0.16 | 0.54 |  |
| SEQ26 | …make gestures or used body language of a sexual nature that embarrassed or offended you? | 5 | 5 | 1 | 0.03 | 0.94 |  | 5 | 1 | 1 | 0.03 | 0.94 |  | 4 | 0.8 | 0.6 | 0.16 | 0.54 |  |
| SEQ27 | …gaze, leer, or ogle at you in a way that made you feel uncomfortable? | 5 | 5 | 1 | 0.03 | 0.94 |  | 4 | 0.8 | 0.6 | 0.16 | 0.54 |  | 5 | 1 | 1 | 0.03 | 0.94 |  |
| SEQ28 | … make you feel you were being bribed with some reward to engage in sexual behaviour? | 5 | 5 | 1 | 0.03 | 0.94 |  | 5 | 1 | 1 | 0.03 | 0.94 |  | 4 | 0.8 | 0.6 | 0.16 | 0.54 |  |
| SEQ29 | …make you feel threatened with some retaliation for not being sexually cooperative? | 5 | 5 | 1 | 0.03 | 0.94 |  | 5 | 1 | 1 | 0.03 | 0.94 |  | 5 | 1 | 1 | 0.03 | 0.94 |  |
| SEQ30 | …make you afraid that you would be handled by him poorly if you did not cooperate sexually? | 5 | 5 | 1 | 0.03 | 0.94 |  | 5 | 1 | 1 | 0.03 | 0.94 |  | 4 | 0.8 | 0.6 | 0.16 | 0.54 |  |
| SEQ31 | …make unwanted attempts to establish a romantic sexual relationship with you? | 5 | 5 | 1 | 0.03 | 0.94 |  | 5 | 1 | 1 | 0.03 | 0.94 |  | 5 | 1 | 1 | 0.03 | 0.94 |  |
| SEQ32 | …continued to ask you for sexual dates, drinks, dinner, even though you said “No”? | 5 | 5 | 1 | 0.03 | 0.94 |  | 5 | 1 | 1 | 0.03 | 0.94 |  | 5 | 1 | 1 | 0.03 | 0.94 |  |
| SEQ33 | …make unwanted attempts to stroke, fondle, or kiss you? | 5 | 5 | 1 | 0.03 | 0.94 |  | 5 | 1 | 1 | 0.03 | 0.94 |  | 5 | 1 | 1 | 0.03 | 0.94 |  |
|  | | S-CVI/Ave | 0.97 | cK*/Ave=0.89 | | | | S-CVI/Ave | 0.86 | Kappa/Ave = 0.75 | | | | S-CVI/Ave | 0.85 | S-CVR/Ave | 0.69 | cK*/Ave | 0.73 |
|  |  | Total Agreement | 29 |  |  |  |  | Total Agreement | 26 |  |  |  |  | Total Agreement | 26 |  | | | |
|  |  | S-CVI/UA | 0.88 |  |  |  |  | S-CVI/UA | 0.79 |  |  |  |  | S-CVI/UA | 0.79 |  |  |  |  |
|  |  | Sum-CVI | 32 |  |  |  |  | Sum-CVI | 28.4 |  |  |  |  | Sum-CVI | 28 |  |  |  |  |
|  |  | CVR | 0.97 |  |  |  |  | CVR | 0.86 |  |  |  |  | CVR | 0.85 |  |  |  |  |

I-CVI (content validity index) = number of experts providing a rating of 3 or 4/number of experts pc (probability of chance occurrence) = [N!/A!(N-A)!] × 0.5N, N = number of experts; A = number of experts agreeing on a rating of 3 or 4 ck* (modified kappa) = (I-CVI-pc)(1-pc) Evaluation criteria for the level of content validity: relationship between I-CVI and k*; excellent validity = I-CVI ≥ 0.78 and k* >0.74 (****); good validity I-CVI < 0.78 and ≥ 0.60 and k* ≤0.74 (***); fair validity I-CVI < 0.6 and ≥ 0.40 and k* ≤0.59 (**); poor validity I-CVI < 0.4 and k*< o.40(*)

**SA 3. Cognitive Protocol**

**Cognitive Interview Protocol**

Hello! My name is Mulugeta Dile. I work at Debre Tabor University. I Thank you so much for your help. Today you will be participating in a particular study on sexual harassment. Its goal is to find better ways to test sexual harassment questionnaire understanding for all hospitality women employees. We need your help creating a well understandable questionnaire. If you decide you do not want to continue, that is your choice, and you are free to stop and go.

Do you have any questions before I begin the instructions?

1. What is this question asking?
2. How did you arrive at your answer?
3. Do you feel this question is easy or not easy to answer?
4. Why do you say that?
5. What would it take for you to say that?
6. What time were you thinking about?

**SA 4. Survey questionnaires**

**Jimma University, Institute of Health Sciences**

**Department of Population and Family Health**

**Survey questionnaire for women working in hospitality workplaces**

**Dear participant,**

**This questionnaire is part of my doctorate research. The study’s intention is about the perception of sexual harassment, the experience of sexual harassment, and coping with sexual harassment on women working in hospitality workplaces. You, as an individual, are selected as a sample of the total population. Hence, you are cordially requested to take part in the survey.**

This study’s primary purpose is to assess women’s perceptions, experiences, and coping mechanisms of sexual harassment in Bahir Dar city’s hospitality workplaces. In addition, researchers wish to find out ways by which sexual harassment in the hospitality workplace can be addressed.

The information you are going to provide is valuable for the success of this research. Therefore, the natural, genuine, and honest response from your personal view and your experience is highly appreciated. There are no predictable risks associated with completing the questionnaire; we will only use the information you provide for academic purposes and keep it strictly confidential. This interview may take approximately 20 to complete. You must not indicate your name to ensure anonymity, but some necessary information about your background helps for a more comprehensive analysis. Your involvement in this survey is voluntary, and you have the right to omit any question or withdraw from answering this interview.

Jimma University, faculty of public health graduate studies, granted permission to conduct this survey. After completing the study, the electronic summary of the research findings will be received upon your request. If you have any research-related queries, you can directly contact me via +251913288238 or e-mail muliedile@gmail.com. You can also get my supervisor, Dr Gurmesa Tura, via +25912061646 or e-mail at gurmesatura@gmail.com and Dr Zewudie Birhanu via +251917025852 or e-mail at zbkoricha@gmail.comzbkoricha@yahoo.com.

By completing the questionnaire, you imply that you have agreed to participate in this research.

Thanks very much for your cooperation!

The researcher

I agree; continue to the next page.

I disagree; continue to the next respondent

Name of data collector ________________________________Signature_____________

Name of supervisor _______________________________Signature_______________

Date of data collected /______/______/_______

1. English Version

| Part 1. The socio-Demographic, Participant personality, and work Characteristics [circle the choice] | | | |
| --- | --- | --- | --- |
| No | Questions | Responses | Remarks |
| SES1 | What is your age in whole years? | /______/ years |  |
| SES2 | What is the participant’s religion? | 1. Orthodox 2. Muslim 3. Protestant 4. Catholic 5. Others (Specify) /_____/ |  |
| SES3 | What is your ethnicity? | 1. Amhara 2. Agew 3. Tigre 4. Oromo 5. Other (specify) /______/ |  |
| SES4 | What is your educational status? | 1. Unable to read and write 2. Able to read and write only 3. Primary education 4. Secondary education 5. College diploma and above |  |
| SES5 | What is your Marital Status? | 1. Single 2. Married 3. Divorced 4. Widowed 5. Separated 6. Cohabiting | If no go to SES7 |
| SES6 | What was your age at your first marriage? | -------- years |  |
| SES7 | To whom do you live (living arrangements)? | 1. Me alone 2. Within Shared house 3. With Family 4. With Friends 5. With Boyfriend 6. With Husband 7. Others (specify) /_____/ |  |
| SES8 | How much is the distance between our home and your workplace? | ---------km |  |
| SES9 | In which organisation are you working? | 1. Cafeteria 2. Hotel 3. Groceries 4. Restaurants |  |
| SES10 | In which part of the organisation are you working (Department)? | 1. Customer reception service 2. Housekeeping 3. Bar 4. Kitchen 5. Restaurant 6. Room Service 7. Others, Specify /______/ |  |
| SES11 | How many years of work experience do you have? | /________/years |  |
| SES12 | What is your employment status? | 1. Full-time  2. Part-time |  |
| SES13 | Who advised you to join this job? | 1. Friends 2. Broker 3. Parents 4. Other (specify) /_______/ |  |
| SES14 | How much is your monthly salary? | /________/ Ethiopian Birr |  |
| SES15 | How much is your average monthly income /including tip? | /____/Ethiopian birr |  |
| SES16 | How do you become interested in working in hospitality workplaces? | 1. I had no option 2. It was due to the tip 3. It is easy to perform 4. Lack of work 5. Other /_____________/ |  |
| SES17 | How much time do you work per day? | /_____________/ hours |  |

| **Part 2. The perception sexual harassment questionnaire for hospitality workplaces (PSHQ_HW):** As a woman working in the hospitality workplace, how do you understand sexual harassment by the supervisors, managers, customers, co-workers, and agents in hospitality workplaces? Legend (check the correct number that applies to each question): 1 = Strongly Disagree 2 = Disagree 3 = Neutral 4 = Agree 5= Strongly Agree | | | | | | |
| --- | --- | --- | --- | --- | --- | --- |
| No | Sexual harassment is… | | | | | |
|  | …preparing for sexual harassment by making inappropriate promises | 5 | 4 | 3 | 2 | 1 |
|  | … preparing women for sexual harassment by providing better career advancement | 5 | 4 | 3 | 2 | 1 |
|  | … an offer of a new job in exchange for sexual advances | 5 | 4 | 3 | 2 | 1 |
|  | …an act of threatening to hurt relatives in exchange for sexual advances | 5 | 4 | 3 | 2 | 1 |
|  | … threatening to complain or falsely accuse about the service being provided to the immediate supervisor unless women agree to sexual favours | 5 | 4 | 3 | 2 | 1 |
|  | … an act of touching sexual sensitive parts while the women are at work | 5 | 4 | 3 | 2 | 1 |
|  | … an act of speaking random sexual jokes to women while she is at work | 5 | 4 | 3 | 2 | 1 |
|  | … a repeated request of women to engage in sexual activities | 5 | 4 | 3 | 2 | 1 |
|  | … an act of emotionally distressing women based on their gender | 5 | 4 | 3 | 2 | 1 |
|  | … an act of poking women with a finger, foot, or pointed object while they are at work | 5 | 4 | 3 | 2 | 1 |
|  | … a request telephone number of women while they are at work | 5 | 4 | 3 | 2 | 1 |
|  | … an act of showing pornographic movies/pictures to women while they are at work | 5 | 4 | 3 | 2 | 1 |
|  | … writing sexual messages on the pay bill to women while they are at work | 5 | 4 | 3 | 2 | 1 |
|  | … an unfair treatment of women because of their gender | 5 | 4 | 3 | 2 | 1 |
|  | … a forcible take of woman for sexual intercourse after they leave their job | 5 | 4 | 3 | 2 | 1 |
|  | … conducting sexual intercourse through force or under threat of injury against the women will | 5 | 4 | 3 | 2 | 1 |
|  | … slapping, kicking, pinching, or insulting women while they refuse to agree to a sexual favour | 5 | 4 | 3 | 2 | 1 |

| **Part 3. The sexual harassment experiences questionnaire for hospitality workplaces (SEQ_HW):** Have you had any of the following actions from your male supervisors/colleagues/clients in this organisation in the last six months? Please indicate 0=never, 1=once/twice, 2=sometimes, 3=often, 4= always | | | | | | |
| --- | --- | --- | --- | --- | --- | --- |
| No | How often did the perpetrator… | | | | | |
|  | …offered you rewards in return for your cooperation in sexual matters? | 0 | 1 | 2 | 3 | 4 |
|  | … mistreat you for your refusal of cooperating with sexual matters? | 0 | 1 | 2 | 3 | 4 |
|  | …tell you sexually explicit jokes? | 0 | 1 | 2 | 3 | 4 |
|  | … sexually assaulted you in public or in private? | 0 | 1 | 2 | 3 | 4 |
|  | …have unwanted sexual conversations with you? | 0 | 1 | 2 | 3 | 4 |
|  | …did the perpetrator repeatedly ask you for sexual dates even after you refused? | 0 | 1 | 2 | 3 | 4 |
|  | …attempts to establish a sexual relationship with you? | 0 | 1 | 2 | 3 | 4 |
|  | …touch you in a way that made you feel uncomfortable? | 0 | 1 | 2 | 3 | 4 |
|  | … violated your personal boundaries? | 0 | 1 | 2 | 3 | 4 |
|  | …target you for rumours of sexual promiscuity? | 0 | 1 | 2 | 3 | 4 |
|  | …insult you by targeting your sexual orientation? | 0 | 1 | 2 | 3 | 4 |
|  | … make unwanted comments about your body, clothing, or way of living? | 0 | 1 | 2 | 3 | 4 |
|  | … show you sexually explicit images or objects that he found unwanted or unpleasant? | 0 | 1 | 2 | 3 | 4 |
|  | …make telephone calls to you or gave you letters with sexual content? | 0 | 1 | 2 | 3 | 4 |
|  | …make unwanted inquiries of sexual services with threats of punishment or sanctions? | 0 | 1 | 2 | 3 | 4 |
|  | … make sexual assaults, attempts of rape, or actual rape? | 0 | 1 | 2 | 3 | 4 |
|  | …unnecessarily expose themselves in front of you? | 0 | 1 | 2 | 3 | 4 |
|  | …threaten you by file a complaint about your service to your supervisor because you refused a sexual request? | 0 | 1 | 2 | 3 | 4 |
|  | …affect you by whistling, calling, or sexually honked at you? | 0 | 1 | 2 | 3 | 4 |
|  | …make unwelcome attempts to draw you into a discussion of sexual matters? | 0 | 1 | 2 | 3 | 4 |
|  | …make offensive remarks about your appearance, body, or sexual activities? | 0 | 1 | 2 | 3 | 4 |
|  | …make gestures or used body language of a sexual nature that embarrassed or offended you? | 0 | 1 | 2 | 3 | 4 |
|  | …gaze, leer, or ogle at you in a way that made you feel uncomfortable? | 0 | 1 | 2 | 3 | 4 |
|  | … make you feel you were being bribed with some reward to engage in sexual behaviour? | 0 | 1 | 2 | 3 | 4 |
|  | …make you feel threatened with some retaliation for not being sexually cooperative? | 0 | 1 | 2 | 3 | 4 |
|  | …make you afraid that you would be handled by him poorly if you did not cooperate sexually? | 0 | 1 | 2 | 3 | 4 |
|  | …make unwanted attempts to establish a romantic sexual relationship with you? | 0 | 1 | 2 | 3 | 4 |
|  | …continued to ask you for sexual dates, drinks, dinner, even though you said “No”? | 0 | 1 | 2 | 3 | 4 |
|  | …make unwanted attempts to stroke, fondle, or kiss you? | 0 | 1 | 2 | 3 | 4 |

| **Part 4. The sexual harassment coping mechanisms for hospitality workplaces (SHCQ_HW):** During the past six months at this organisation, have you been in a situation where any of your male supervisors/ co-workers/customers harassed you and responded in the following ways? Please indicate 0=never, 1=once/twice, 2=sometimes, 3=often, 4= always | | | | | | |
| --- | --- | --- | --- | --- | --- | --- |
| No | How often have you tried to… | | | | | |
|  | …file a formal complaint about sexual harassment? | 0 | 1 | 2 | 3 | 4 |
|  | …report the perpetrator of sexual harassment? | 0 | 1 | 2 | 3 | 4 |
|  | …talk with supervisors, managers, and unions about the event of sexual harassment? | 0 | 1 | 2 | 3 | 4 |
|  | …tell the perpetrator that you do not like sexual harassment? | 0 | 1 | 2 | 3 | 4 |
|  | …forget about the event of sexual harassment? | 0 | 1 | 2 | 3 | 4 |
|  | …play games or engage in outdoor activities to forget about sexual harassment events? | 0 | 1 | 2 | 3 | 4 |
|  | …watching TV more than usual to forget about the event of sexual harassment? | 0 | 1 | 2 | 3 | 4 |
|  | …going to a friend for advice on how to change the issue of sexual harassment? | 0 | 1 | 2 | 3 | 4 |
|  | …get sympathy and understanding from friends who have had the same problem? | 0 | 1 | 2 | 3 | 4 |
|  | …talk to people about the situation because talking about it makes you feel better? | 0 | 1 | 2 | 3 | 4 |
|  | …seek reassurance from those who know you best? | 0 | 1 | 2 | 3 | 4 |
|  | …set some goals for yourself to deal with the issue of sexual harassment? | 0 | 1 | 2 | 3 | 4 |
|  | …carefully plan a course of action rather than acting on impulse? | 0 | 1 | 2 | 3 | 4 |
|  | …decided not to participate in hospitality work? | 0 | 1 | 2 | 3 | 4 |
|  | …pray while experiencing sexual harassment? | 0 | 1 | 2 | 3 | 4 |
|  | …changed your job frequently because of sexual harassment? | 0 | 1 | 2 | 3 | 4 |
|  | …bring the perpetrator to justice? | 0 | 1 | 2 | 3 | 4 |
|  | …consult a health care provider because of sexual harassment? | 0 | 1 | 2 | 3 | 4 |
|  | …consult a psychologist because of the sexual harassment that you face? | 0 | 1 | 2 | 3 | 4 |
|  | … negotiate with the perpetrator? | 0 | 1 | 2 | 3 | 4 |
|  | …discriminate against the perpetrators? | 0 | 1 | 2 | 3 | 4 |
|  | …reject the request for sexual harassment? | 0 | 1 | 2 | 3 | 4 |
|  | …confront the perpetrator? | 0 | 1 | 2 | 3 | 4 |
|  | …tolerate sexual harassment? | 0 | 1 | 2 | 3 | 4 |
|  | …keep silent to respond to sexual harassment? | 0 | 1 | 2 | 3 | 4 |
|  | …ignore sexual harassment? | 0 | 1 | 2 | 3 | 4 |
|  | …accept sexual harassment? | 0 | 1 | 2 | 3 | 4 |

1. Amharic Version

ጅማ ዩኒቨርሲቲ የጤና ሳይንስ ተቋም

የስነ-ህዝብ እና የቤተሰብ ጤና ትምህርት ክፍል

በእንግዳ ማረፊያ ቦታዎች ውስጥ ለሚሠሩ ሴቶች የተዘጋጀ የዳሰሳ ጥናት መጠይቅ

ውድ ተሳታፊዎች ፣

መግቢያ፥- ስሜ -----------------እባላለሁ፡፡ በአሁኑ ሰዓት በጅማ ዩኒቨርስቲ ውስጥ እገኛለሁ፡፡ ይህ መጠይቅ በጅማ ዩኒቨርስቲ የዶክቶሬት ትምህርቴ የጥናት አካል ነው፡፡ የጽሑፉ የትኩረት አቅጣጫ በባህር ዳር ከተማ አስተዳደር ውስጥ በእንግዳ ማረፊያ የሥራ ቦታዎች ውስጥ በሚሠሩ ሴቶች ላይ ያለውን የወሲባዊ ትንኮሳ ግንዛቤ፣ ስፋት፣ ተያያዥነት ያላቸው ምክንያቶች እና ተጽዕኖዎች ናቸው፡፡ እርስዎ ከጠቅላላው ህዝብ እንደ ናሙና ተመርጠዋል፡፡ ስለሆነም በዳሰሳ ጥናቱ እንዲሳተፉ በአክብሮት ተጋብዘዋል፡፡

የጥናቱ ዓላማ፥- የዚህ ጥናት ዋና ዓላማ በባህር ዳር ከተማ መስተንግዶ ውስጥ በሚሠሩ ሴቶች ላይ ያለውን የወሲባዊ ትንኮሳ ግንዛቤዎችን፣ የወሲባዊ ትንኮሳ ትንታኔዎችን፣ መጠኑን፣ ተጓዳኝ ሁኔታዎችን መገምገም ነው ፡፡ ተመራማሪዎቹ በእንደዚህ ያሉ ስብስቦች ውስጥ ለወሲባዊ ትንኮሳ አስተዋፅኦ የሚያደርጉትን ደረጃዎች እና ምክንያቶች በመገምገም በእንግዳ መስተንግዶ ኢንዱስትሪዎች ውስጥ የወሲብ ትንኮሳ የሚስተካከሉበትን መንገድ ይፈልጋሉ ፡፡

ሂደቶች፥- የሚያቀርቡት መረጃ ለዚህ የምርምር ስኬት ጠቃሚ ነው ፡፡ ስለዚህ ከግል እይታዎ እና ተሞክሮዎ በመነሳት የሚሰጡት ግልፅ ፣ እውነተኛ እና ሀቀኛ ምላሽ በጣም ጠቃሚ ነው፡፡ መጠይቁን ከማጠናቀር ጋር የተዛመዱ ሊታዩ የሚችሉ አደጋዎች የሉም ፣ ያቀረቡት መረጃ ለአካዳሚክ ዓላማ ብቻ ጥቅም ላይ የሚውል ሲሆን ምስጢራዊነቱ የተጠበቀ ይሆናል፡፡ ይህን ቃለ መጠይቅ ለማጠናቀቅ ከ 10 እስከ 20 ደቂቃዎችን ሊወስድ ይችላል ፡፡

ስጋት እና ምቾት፥- ይህ ጥናት በሥራ ቦታ ስለ ወሲባዊ ትንኮሳ ስለሚናገር ለአንዳንድ ጥያቄዎች መልስ ለመስጠት ምቾት ላይሰማዎት ይችል ይሆናል ፡፡ ከፈለጉ ለቃለ መጠይቅዎ አንድ ደጋፊ የሆነ ሰው ይዘው መምጣት ይችላሉ ፡፡ በተጨማሪም ፣ ከጠየቁን ቃለ መጠይቁን እና እናቆማለን ፡፡

ጥቅሞች፥- በእንግዳ ተቀባይነት የሥራ ቦታ ውስጥ ለሠራተኞቹ የሚሰጠውን ሥልጠና በሚሰጥ ጥናት ላይ አስተዋጽኦ ያደርጋሉ ፡፡ ስምዎ እና የስራ ቦታዎ ጥቅም ላይ አይውሉም ፣ እና በአስተማማኝ ሁኔታ ይቀመጣሉ። እኛ ወደ እርስዎ ስለምንመጣ ፣ የሚያጋጥምዎት ወጪ ጊዜዎ ብቻ ነዉ።

ምስጢራዊነት፥- የማንነትዎን መደበቅ ለማረጋገጥ ስምዎን እንዲያመለክቱ አይጠየቁም፡፡ ነገር ግን ስለ ዳራዎ አንዳንድ መሰረታዊ መረጃዎችን ማወቅ ለበለጠ አጠቃላይ ትንታኔ ይረዳል ፡፡ በዚህ የዳሰሳ ጥናት ውስጥ ያለዎት ተሳትፎ በፈቃደኝነት ነው፡፡ እናም ከፈለጉ ማንኛውንም ጥያቄ የመተው ወይም ለዚህ ቃለ ምልልስ መልስ ያለመስጠት መብት አልዎት ፡፡

የመቃወም ወይም የመተው መብት፥- ጥናቱ ከተጠናቀቀ በኋላ የጥናቱ ግኝቶች የኤሌክትሮኒክ ማጠቃለያ ይጋራሉ፡፡ ይህንን ቃለ መጠይቅ የማካሄድ ፈቃድ የተሰጠው በጂማ ዩኒቨርሲቲ የጤና ኢንስቲትዩት የህዝብ ጤና ምረቃ ጥናቶች ፋኩልቲ ነው፡፡ ማንኛውንም ምርምር-ነክ መጠይቅ ካለዎት በቀጥታ በ 251913288238 ወይም በኢሜል muliedile@gmail.com በኩል አነጋግርዎታለሁ ወይም ተቆጣጣሪዎቼን በስልክ ቁጥር 25912061646 እና gurmesatura@gmail.com ወይም 251917025852 እና zbkoricha @ yahoo.com ላይ ማግኘት ይችላሉ፡፡

መጠይቁን በማጠናቀቅዎ በዚህ ምርምር ውስጥ ለመሳተፍ መስማማትዎን ያሳያል ፡፡ ስለ ትብብርዎ በቅድሚያ እናመሰግናለን!

ተመራማሪው

እስማማለሁ ፣ ወደሚቀጥለው ገጽ ይቀጥሉ ፡፡

ካልተስማሙ ወደሚቀጥለው ምላሽ ሰጪ ይቀጥሉ

የመረጃ ሰብሳቢው ስም ________________________________ ፊርማ _______

የተቆጣጣሪ ስም _______________________________ ፊርማ _______

የተሰበሰበበት ቀን / ______ / ______ / ______

| **ክፍል 1.** የስነ-ሕዝባዊ ፣ የተሣታፊ ስብዕና ፣ እና የሥራ ባህሪ መረጃ ጠቋሚ [ምርጫውን ይክበቡ] | | | | | | | | | | | | | | | |
| --- | --- | --- | --- | --- | --- | --- | --- | --- | --- | --- | --- | --- | --- | --- | --- |
| ቁጥር | | | ጥያቄ | ምላሽ | | | ማስታወሻዎች | | | | | | | | |
| SES1 | | | ዕድሜዎ ስንት ነዉ? | /_______________________/ | | |  | | | | | | | | |
| SES2 | | | ሃይማኖትዎ ምንድን ነው? | 1. ኦርቶዶክስ  2. ሙስሊም  3. ፕሮቴስታንት  4. ካቶሊክ  5. ሌሎች (ይግለጹ) / -------------- | | |  | | | | | | | | |
| SES3 | | | ጎሳዎ ምንድ ነው? | 1) አማራ  2) አገዉ  3) ትግሬ  4) ኦሮሞ  5) ሌላ (ይግለጹ) / --------------- | | |  | | | | | | | | |
| SES4 | | | የትምህርት ደረጃዎ ምን ያህል ነው? | 1. ማንበብ እና መፃፍ አልችልም  2. ማንበብ እና ለመጻፍ ብቻ  3. የመጀመሪያ ደረጃ ትምህርት  4. ሁለተኛ ደረጃ ትምህርት  5. የኮሌጅ ዲፕሎማ እና ከዚያ በላይ | | |  | | | | | | | | |
| SES5 | | | የጋብቻ ሁኔታዎ ምንድ ነው? | 1. አላገባሁም  2. አግብቻለሁ  3. ተፋትቻለሁ  4. ባለቤቴ ሞቶብኛል  5. ተለያይቸ ነዉ የምኖረዉ  6. ሳላገባ ከጓደኛዬ ጋር አብሬ እኖራለሁ | | | ያላገቡ ከሆኑ ወደ ቁጥር ሰባት ይዝለሉ ፡፡ | | | | | | | | |
| SES6 | | | በመጀመሪያው ጋብቻዎ ላይ ዕድሜዎ ስንት ነበር? | /_________________/ አመት | | |  | | | | | | | | |
| SES7 | | | ከማን ጋር ነው የሚኖሩት? | 1. እኔ ብቻዬን  2. በጋራ ቤት ውስጥ  3. ከቤተሰብ ጋር  4. ከጓደኞች ጋር  5. ከወንድ ጓደኛ ጋር  6. ከባለቤት ጋር  7. ሌሎች (ይግለጹ) /----------------- | | |  | | | | | | | | |
| SES8 | | | የመኖሪያ ቤትዎ ከሥራ ቦታዎ ምን ያህል ይርቃል? | / ______ / ኪሎ ሜትር | | |  | | | | | | | | |
| SES9 | | | የሚሰሩት በየትኛው ድርጅት ውስጥ ነው? | 1. ካፌቴሪያ  2. ሆቴል  3. ግሮሰሪ  4. ምግብ ቤቶች | | |  | | | | | | | | |
| SES10 | | | እርስዎ በየትኛው የድርጅቱ ክፍል እየሰሩ ነው? | 1. የደንበኞች አቀባበል አገልግሎት  2. የስራ ቦታ ስነ-ዉበት ጥበቃ  3. ቡና ቤት  4. ወጥ ቤት  5. ሬስቶራንት  6. ክፍል አገልግሎት  7. ሌሎች (ይግለጹ)------------------------- | | |  | | | | | | | | |
| SES11 | | | የሥራ ሁኔታዎ ምንድን ነው? | 1. ሙሉ ቀን  2. የተወሰነ ሰዓት | | |  | | | | | | | | |
| SES12 | | | ስንት ዓመት የስራ ልምድ አለዎት? | ----------ዓመት | | |  | | | | | | | | |
| SES13 | | | በዚህ ሥራ እንዲካፈሉ ማን መከረዎት? | 1. ጓደኞች  2. ደላላ  3. ወላጆች  4. በራስሽ ተነሳስተሽ  4. ሌሎች ---------------------------- | | |  | | | | | | | | |
| SES14 | | | ወርሃዊ ደሞዝዎ ስንት ነው? | /________/ የኢትዮጵያ ብር | | |  | | | | | | | | |
| SES15 | | | ጉርሻን ጨምሮ የእርስዎ አማካይ ወርሃዊ ገቢ ስንት ነው? | /________/ የኢትዮጵያ ብር | | |  | | | | | | | | |
| SES16 | | | በእንግዳ ተቀባይነት ቦታ ለመሥራት ፍላጎት ያደረብዎ እንዴት ነው? | 1. ምንም አማራጭ አልነበረኝም  2. ጉርሻ ምክንያት ነበር  3. በቀላሉ ማከናወን ይቻላል  4. የስራ አለመኖር  5. ሌላም /_______________ | | |  | | | | | | | | |
| SES17 | | | በየቀኑ ምን ያህል ጊዜ ይሰራሉ? | /_________________/ ስዓት | | |  | | | | | | | | |
| **ክፍል 2:** በመስተንግዶ የሥራ ቦታዎች የሚሰሩ ሴቶች የወሲብ ትንኮሳ ግንዛቤ መጠይቅ (PSHQ_HW) ፥ እንግዳ ተቀባይ በሆነ የሥራ ቦታ ውስጥ እንደምትሠራ ሴት በቅርብ የበሥራ ኃላፊዎች ፣ በሥራ አስኪያጆች ፣ በደንበኞች ፣ በሥራ ባልደረቦች እና ብደላሎች የሚደረግን የወሲብ ትንኮሳ እንዴት ትገነዘቢዋለሽ? (ለእያንዳንዱ ጥያቄ የሚሠራውን ትክክለኛ ቁጥር ያመልከቱ) 1 = በጣም አልስማማም 2 = አልስማማም፣ 3 = ገለልተኛ፣ 4 = እስማማለሁ፣ 5= በጣም እስማማለሁ | | | | | | | | | | | | | | | |
| ቁጥር | መገለጫ | | | | | | | | | | | | | | |
|  | ተገቢ ያልሆነ የተስፋ ቃል በመስጠት ለወሲባዊ ትንኮሳ የማዘጋጀት ሂደት ነው | | | | | 5 | | | 4 | | 3 | | 2 | | 1 |
|  | ሻል ያለ የስራ እድገት በመስጠት ሴቶችን ለወሲባዊ ትንኮሳ የማዘጋጀት ድርጊት ነው | | | | | 5 | | | 4 | | 3 | | 2 | | 1 |
|  | ለሴቶችን አዲስ ሥራ በመስጠት ለወሲባዊ ትንኮሳ የማዘጋጀት ተግባር ነው | | | | | 5 | | | 4 | | 3 | | 2 | | 1 |
|  | ለወሲባዊ ግንኙነት ካልተስማሙ ዘመዶቻቸውን ለመጉዳት የማስፈራራት ተግባር ነው | | | | | 5 | | | 4 | | 3 | | 2 | | 1 |
|  | ሴቶች ለወሲባዊ ግንኙነት ካልተስማሙ በቀር ለቅርብ ተቆጣጣሪው ስለሚሰጠው አገልግሎት ቅሬታ ለማቅረብ ወይም በሐሰት የማስፈራራት ተግባር ነው፡፡ | | | | | 5 | | | 4 | | 3 | | 2 | | 1 |
|  | ሴቶቹ በስራ ላይ እያሉ ወሲባዊ ስሜትን የሚነኩ ክፍሎችን የመነካካት ድርጊት ነው | | | | | 5 | | | 4 | | 3 | | 2 | | 1 |
|  | በሥራ ላይ ሳለች በዘፈቀደ ወሲባዊ ቀልዶችን ለሴቶች የመናገር ድርጊት ነው | | | | | 5 | | | 4 | | 3 | | 2 | | 1 |
|  | ሴቶች በፆታዊ ግንኙነት እንዲሳተፉ ተደጋጋሚ ጥያቄ የመጠየቅ ሂደት ነው | | | | | 5 | | | 4 | | 3 | | 2 | | 1 |
|  | በጾታቸው ላይ ተመስርተው የሴቶችን ስሜት የመንካት ተግባር ነው | | | | | 5 | | | 4 | | 3 | | 2 | | 1 |
|  | ሴቶችን በስራ ላይ እያሉ በጣት ፣ በእግር ወይም በመጠቆሚያ ነገር የመልከፍ ድርጊት ነው | | | | | 5 | | | 4 | | 3 | | 2 | | 1 |
|  | በስራ ላይ እያሉ የሴቶችን የስልክ ቁጥር ጥያቄ የመጠየቅ ሂደት ነውነው | | | | | 5 | | | 4 | | 3 | | 2 | | 1 |
|  | ሴቶችን በስራ ላይ እያሉ የወሲብ ፊልሞችን / ምስሎችን ማሳየት ነው | | | | | 5 | | | 4 | | 3 | | 2 | | 1 |
|  | ለሴቶች በሥራ ላይ እያሉ በክፍያ ደረሰኝ ላይ የወሲብ መልዕክቶችን መፃፍ ነው | | | | | 5 | | | 4 | | 3 | | 2 | | 1 |
|  | በጾታቸው ምክንያት በሴቶች ላይ የሚደረግ ኢ-ፍትሃዊ አያያዝ ነው | | | | | 5 | | | 4 | | 3 | | 2 | | 1 |
|  | ከሥራቸው ሲወጡ ጠብቆ የግብረ ሥጋ ግንኙነት ለመፈፀም በግዳጅ የመውሰድ ተግባር ነው | | | | | 5 | | | 4 | | 3 | | 2 | | 1 |
|  | በሴቶች ላይ የጉዳት ወይም የጉዳት ሥጋት በማድረስ የግብረ ሥጋ ግንኙነት ማከናወን ነው | | | | | 5 | | | 4 | | 3 | | 2 | | 1 |
|  | ለወሲባዊ ትንኮሳ ፈቃደኛ ባለመሆናቸው ሴቶችን የመምታት ፣ የመቆንጠጥ ወይም የመሳደብ ተግባር ነው | | | | | 5 | | | 4 | | 3 | | 2 | | 1 |
| **ክፍል 3:** በእንግዳ ማረፊያ ቦታ የመስተንግዶ ስራ በሚሰሩ ሴቶች ላይ የሚፈጸሙ የወሲባዊ ትንኮሳ ተሞክሮዎች: ባለፉት 12 ወራት ውስጥ በዚህ ድርጅት ውስጥ ካሉ ወንድ ተቆጣጣሪዎችዎ / የሥራ ባልደረቦችዎ / ደንበኞችዎ ወይም ከደላሎች የሚከተሉት ድርጊቶች አጋጥመውዎት ያዉቃሉ? (ለእያንዳንዱ ጥያቄ የሚሠራውን ትክክለኛ ቁጥር ያመልከቱ) 0 = በፍጹም 1 = አንድ /ሁለት ጊዜ 2 = አንዳንዴ 3 =ብዙ ጊዜ 4= ሁልጊዜ | | | | | | | | | | | | | | | |
| ቁጥር | | ጥያቄዎች /መግለጫዎች/ | | | | | | | | | | | | | |
|  | | በወሲባዊ ጉዳዮች ላይ ላደረጉት ትብብር በምላሹ ሽልማት ተሰጥቶዎታል? | | | 0 | | | 1 | | 2 | | 3 | | 4 | |
|  | | ከወሲባዊ ጉዳዮች ጋር ለመተባበር ፈቃደኛ ባለመሆንዎ በደል ደርሶብዎታል? | | | 0 | | | 1 | | 2 | | 3 | | 4 | |
|  | | ብዙውን ጊዜ ግልጽ ወሲባዊ ቀልዶች ይነግርዎታል? | | | 0 | | | 1 | | 2 | | 3 | | 4 | |
|  | | በአደባባይ ወይም በግል ወሲባዊ ትንኮሳ ደርሶብዎታል? | | | 0 | | | 1 | | 2 | | 3 | | 4 | |
|  | | ከእርስዎ ጋር የማይፈለጉ ወሲባዊ ውይይቶችን ይደረግ ነበር? | | | 0 | | | 1 | | 2 | | 3 | | 4 | |
|  | | እርስዎ ፈቃደኛ ካልሆኑ በኋላም ቢሆን የግብረ ሥጋ ግንኙነት ቀኖችን ተደጋግሞ ተጠይቀዋል? | | | 0 | | | 1 | | 2 | | 3 | | 4 | |
|  | | ከእርስዎ ጋር ወሲባዊ ግንኙነት ለመመሥረት ሙከራ ነበር? | | | 0 | | | 1 | | 2 | | 3 | | 4 | |
|  | | ምቾት እንዳይሰማዎት በሚያደርግ ሁኔታ ተነክተዋል? | | | 0 | | | 1 | | 2 | | 3 | | 4 | |
|  | | ከእርስዎ ጋር አላስፈላጊ ግንኙነት አድርገዋል ወይንስ የግል ድንበሮችዎን ጥሰዋል? | | | 0 | | | 1 | | 2 | | 3 | | 4 | |
|  | | የወሲባዊ ብልግና ወሬ እርስዎን ዒላማ አደረጎዎት ነበር? | | | 0 | | | 1 | | 2 | | 3 | | 4 | |
|  | | የፆታ ግንዛቤዎን ዒላማ በማድረግ ተሰድበዋል? | | | 0 | | | 1 | | 2 | | 3 | | 4 | |
|  | | ስለ ሰውነትዎ ፣ ስለ ልብስዎ ወይም ስለ አኗኗርዎ የማይፈለጉ አስተያየቶችን ተሰጥቷል? | | | 0 | | | 1 | | 2 | | 3 | | 4 | |
|  | | የማይፈለጉ ወይም ደስ የማይል የወሲብ ግልፅ ምስሎችን ወይም ዕቃዎችን አሳይዎታል? | | | 0 | | | 1 | | 2 | | 3 | | 4 | |
|  | | በስልክ ጥሪ ያደርግልዎታል ወይንስ ወሲባዊ ይዘት ያላቸውን ደብዳቤዎች ይሰጥዎታል? | | | 0 | | | 1 | | 2 | | 3 | | 4 | |
|  | | በቅጣት ማስፈራሪያ ወይም ማዕቀብ በማስፈራራት ያልተፈለጉ ጥያቄዎችን / የወሲብ አገልግሎቶችን ተጠይቀዋል? | | | 0 | | | 1 | | 2 | | 3 | | 4 | |
|  | | የአስገድዶ መድፈር ሙከራዎችን ወይም በትክክል ተገደዉ ተደፍረዋል? | | | 0 | | | 1 | | 2 | | 3 | | 4 | |
|  | | ሳያስፈልግ ከፊትዎ ሆነው ራሳቸውን አጋልጠዋል? | | | 0 | | | 1 | | 2 | | 3 | | 4 | |
|  | | ወሲባዊ ጥያቄን ባለመቀበልዎ ለሥራ ተቆጣጣሪዎ ስለ አገልግሎትዎ ቅሬታ በማቅረብ ተስፈራራተዋል? | | | 0 | | | 1 | | 2 | | 3 | | 4 | |
|  | | በፉጨት ፣ በመደወል ወይም በወሲብ በመንካት በርስዎ ላይ ተጽዕኖ አሳድረዋል? | | | 0 | | | 1 | | 2 | | 3 | | 4 | |
|  | | እርስዎን ወደ ወሲባዊ ጉዳዮች ውይይት ለመሳብ ያልተፈለጉ ሙከራዎችን ትደርገዋል? | | | 0 | | | 1 | | 2 | | 3 | | 4 | |
|  | | ስለ መልክዎ ፣ ስለ ሰውነትዎ ወይም ስለ ወሲባዊ እንቅስቃሴዎችዎ አፀያፊ አስተያየቶችን ተሰጥተዋል? | | | 0 | | | 1 | | 2 | | 3 | | 4 | |
|  | | እርስዎን ቅር ያሰኘዎትን የወሲብ ተፈጥሮ ምልክቶች ወይም አካላዊ መግለጫ ተጠቅሟል? | | | 0 | | | 1 | | 2 | | 3 | | 4 | |
|  | | ምቾት እንዲሰማዎት በማያደርግ ሁኔታ አይቶዎታል? | | | 0 | | | 1 | | 2 | | 3 | | 4 | |
|  | | በወሲባዊ ባህሪ ለመሳተፍ በተወሰነ ሽልማት እንደተደለሉ እንዲሰማዎት ተደርግዋል? | | | 0 | | | 1 | | 2 | | 3 | | 4 | |
|  | | ወሲባዊ ተባባሪ ባለመሆንዎ በበቀል እርምጃዎች የማስፈራራት ስሜት እንዲሰማዎ ተደርግዋል? | | | 0 | | | 1 | | 2 | | 3 | | 4 | |
|  | | በግብረ ሥጋ ግንኙነት ካልተባበሩ በደፈናው በእሱ እንደሚይዙት ያስፈራዎታል? | | | 0 | | | 1 | | 2 | | 3 | | 4 | |
|  | | ከእርስዎ ጋር የፍቅር ወሲባዊ ግንኙነት ለመመሥረት የማይፈለጉ ሙከራዎች ተደርገዋል? | | | 0 | | | 1 | | 2 | | 3 | | 4 | |
|  | | “አይሆንም” ቢሉም ወሲባዊ ቀናትን ፣ መጠጦችን ፣ እራት ወዘተ በተደጋጋሚ ተጠይቀዋል? | | | 0 | | | 1 | | 2 | | 3 | | 4 | |
|  | | ለመደብደብ ፣ ለመወደድ ወይም ለመሳም የማይፈለጉ ሙከራዎችን አጋጥሞዎታል? | | | 0 | | | 1 | | 2 | | 3 | | 4 | |

| **ክፍል 4:** በእንግዳ ማረፊያ ቦታ የመስተንግዶ ስራ በሚሰሩ ሴቶች ላይ የሚፈጸሙ የወሲባዊ ትንኮሳ ተሞክሮዎች መቋቋሚያ መንገዶች: በዚህ ድርጅት ውስጥ ባለፉት 12 ወራት ውስጥ ከወንድ ተቆጣጣሪዎ / የሥራ ባልደረባዎ / ደንበኛዎ መካከል ወሲባዊ ጥቃት የሚፈጽሙብዎት እና በሚከተሉት መንገዶች ምላሽ የሚሰጡበት ሁኔታ ውስጥ ነበሩ? (ለእያንዳንዱ ጥያቄ የሚሠራውን ትክክለኛ ቁጥር ያመልከቱ) 0 = በፍጹም፣ 1 = አንድ /ሁለት ጊዜ ፣ 2 = አንዳንዴ፣ 3 =ብዙ ጊዜ ፣4= ሁልጊዜ | | | | | | |
| --- | --- | --- | --- | --- | --- | --- |
| ቁጥር | ጥያቄዎች /መግለጫዎች/ |  | | | | |
|  | ስለ ወሲባዊ ትንኮሳ መደበኛ ቅሬታ ለማቅረብ ሞክረዋል? | 0 | 1 | 2 | 3 | 4 |
|  | የወሲብ ትንኮሳ ፈፃሚውን ሪፖርት ለማድረግ ሞክረዋል? | 0 | 1 | 2 | 3 | 4 |
|  | ስለ ወሲባዊ ትንኮሳ ክስተት ከተቆጣጣሪዎች ፣ ሥራ አስኪያጆችና የሠራተኛ ማኅበራት ጋር ለመነጋገር ሞክረዋል? | 0 | 1 | 2 | 3 | 4 |
|  | ለወሲባዊ ተንኳሹ ወሲባዊ ጥቃትን እንደማይወዱ ለመንገር ሞክረዋል? | 0 | 1 | 2 | 3 | 4 |
|  | ስለ ወሲባዊ ትንኮሳ ክስተት ለመርሳት ሞክረዋል? | 0 | 1 | 2 | 3 | 4 |
|  | ስለ ወሲባዊ ትንኮሳ ክስተት ለመርሳት ለመጫወት ወይም ከቤት ውጭ ሥራ ለመሳተፍ ሞክረው ያውቃሉ? | 0 | 1 | 2 | 3 | 4 |
|  | ስለ ወሲባዊ ትንኮሳ ክስተት ለመርሳት ከተለመደው በላይ ቴሌቪዥን ለመመልከት ሞክረዋል? | 0 | 1 | 2 | 3 | 4 |
|  | ሁኔታውን እንዴት መለወጥ እንደሚቻል ምክር ለማግኘት ወደ ጓደኛዎ ለመሄድ ሞክረዋል? | 0 | 1 | 2 | 3 | 4 |
|  | ተመሳሳይ ችግር ካጋጠማቸው ጓደኞች ርህራሄ እና ግንዛቤ ለማግኘት ሞክረዋል? | 0 | 1 | 2 | 3 | 4 |
|  | ስለ ሁኔታው ​​ማውራት ጥሩ ስሜት እንዲሰማዎት ስለሚያደርግ ​​ከሰዎች ጋር ለመነጋገር ሞክረዋል? | 0 | 1 | 2 | 3 | 4 |
|  | በደንብ ከሚያውቁዎት ሰዎች ማጽናኛ ለማግኘት ሞክረዋል? | 0 | 1 | 2 | 3 | 4 |
|  | ሁኔታውን ለመቋቋም አንዳንድ ግቦችን ለራስዎ አውጥተዋል? | 0 | 1 | 2 | 3 | 4 |
|  | በግብታዊነት ከመንቀሳቀስ ይልቅ የድርጊት ጎዳና በጥንቃቄ ለማቀድ ሞክረዋል? | 0 | 1 | 2 | 3 | 4 |
|  | በመስተንግዶ የሥራ ቦታ ሥራ ላለመሳተፍ ወስነዋል? | 0 | 1 | 2 | 3 | 4 |
|  | ወሲባዊ ትንኮሳ እያጋጠመዎት ለመጸለይ ሞክረዋል? | 0 | 1 | 2 | 3 | 4 |
|  | በወሲባዊ ትንኮሳ ምክንያት ሥራዎን በተደጋጋሚ ቀይረዋል? | 0 | 1 | 2 | 3 | 4 |
|  | ወሲባዊ ተንኳሹን ለፍርድ ለማቅረብ ሞክረዋል? | 0 | 1 | 2 | 3 | 4 |
|  | በወሲባዊ ትንኮሳ ምክንያት የጤና ባለሙያን ለማማከር ሞክረዋል? | 0 | 1 | 2 | 3 | 4 |
|  | በሚያጋጥሙዎት ወሲባዊ ትንኮሳ ምክንያት የሥነ ልቦና ባለሙያውን ለማማከር ሞክረዋል? | 0 | 1 | 2 | 3 | 4 |
|  | ከወሲባዊ ተንኳሹ ጋር ለመደራደር ሞክረዋል? | 0 | 1 | 2 | 3 | 4 |
|  | ወሲባዊ ተንኳሹ ላይ አድልዎ ለማድረግ ሞክረዋል? | 0 | 1 | 2 | 3 | 4 |
|  | የወሲብ ትንኮሳ ጥያቄን ውድቅ ለማድረግ ሞክረዋል? | 0 | 1 | 2 | 3 | 4 |
|  | ወሲባዊ ተንኳሹን ለመጋፈጥ ሞክረዋል? | 0 | 1 | 2 | 3 | 4 |
|  | ወሲባዊ ትንኮሳዎችን ለመታገስ ሞክረዋል? | 0 | 1 | 2 | 3 | 4 |
|  | ለወሲባዊ ትንኮሳ ምላሽ ከመስጠት ለመታቀብ ሞክረዋል? | 0 | 1 | 2 | 3 | 4 |
|  | ወሲባዊ ጥቃትን ችላ ለማለት ሞክረዋል? | 0 | 1 | 2 | 3 | 4 |
|  | ወሲባዊ ትንኮሳን ለመቀበል ሞክረዋል? | 0 | 1 | 2 | 3 | 4 |

**SA 5. Participant’s characteristics and working organisation related characteristics**

| Variables | | Frequency (%) |
| --- | --- | --- |
|  | Orthodox | 304 (88.1) |
| Religion | Muslim | 6 (1.7) |
|  | Protestant | 28 (8.1) |
|  | catholic | 7 (2) |
|  | Amhara | 300 (87) |
|  | Agew | 14 (4.1) |
| Ethnicity | Tigre | 9 (2.6) |
|  | Oromo | 18 (5.2) |
|  | Others | 4 (1.2) |
|  | Unable to read and write | 13 (3.8) |
|  | Able to write and read | 29 (8.4) |
| Educational status | Primary education | 70 (20.3) |
|  | Secondary education | 124 (35.9) |
|  | College diploma and above | 109 (31.6) |
|  | Single | 230 (66.7) |
|  | Married | 68 (19.7) |
| Marital status | Divorced | 26 (7.5) |
|  | Widowed | 4 (1.2) |
|  | Separated | 8 (2.3) |
|  | Cohabited | 9 (2.6) |
|  | Alone | 139 (40.3) |
|  | Shared house | 16 (4.6) |
| Living arrangement | Family | 62 (18) |
|  | Friends | 58 (16.8) |
|  | Boyfriend | 18 (5.2) |
|  | Husband | 52 (15.1) |
|  | Cafeteria | 59 (17.1) |
| Organisation | Hotel | 160 (46.4) |
|  | Grocery | 78 (22.6) |
|  | Restaurant | 48 (13.9) |
|  | Reception | 85 (24.6) |
|  | Housekeeping | 29 (8.4) |
| Working department | Kitchen | 50 (14.5) |
|  | Room service | 33 (9.6) |
|  | Waitering | 148 (42.9) |
| Employment status | Full time | 143 (41.4) |
|  | Part-time | 202 (58.6) |
|  | Friends | 66 (19.1) |
| Initiated by | Broker | 89 (25.8) |
|  | Parents | 14 (4.1) |
|  | Self | 176 (51) |
|  | Lack of options | 112 (32.5) |
|  | The tip | 56 (16.2) |
| Reason of engagement in HW | Easiness of the work | 44 (12.8) |
|  | Lack of other jobs | 123 (35.7) |
|  | Others | 10 (2.9) |

**SA 6. Pearsons product-moment correlations for concurrent validity**

1. Perceptions

| Correlations | | | | | | | | | | | | | |
| --- | --- | --- | --- | --- | --- | --- | --- | --- | --- | --- | --- | --- | --- |
|  | | PerTot | PSHQ_HW1 | PSHQ_HW2 | PSHQ_HW3 | PSHQ_HW4 | PSHQ_HW5 | PSHQ_HW7 | PSHQ_HW8 | PSHQ_HW6 | PSHQ_HW15 | PSHQ_HW16 | PSHQ_HW17 |
| PerTot | Pearson Correlation | 1 |  |  |  |  |  |  |  |  |  |  |  |
|  | Sig. (2-tailed) |  |  |  |  |  |  |  |  |  |  |  |  |
|  | N | 345 |  |  |  |  |  |  |  |  |  |  |  |
| PSHQ_HW1 | Pearson Correlation | .749^**^ | 1 |  |  |  |  |  |  |  |  |  |  |
|  | Sig. (2-tailed) | .000 |  |  |  |  |  |  |  |  |  |  |  |
|  | N | 345 | 345 |  |  |  |  |  |  |  |  |  |  |
| PSHQ_HW2 | Pearson Correlation | .806^**^ | .704^**^ | 1 |  |  |  |  |  |  |  |  |  |
|  | Sig. (2-tailed) | .000 | .000 |  |  |  |  |  |  |  |  |  |  |
|  | N | 345 | 345 | 345 |  |  |  |  |  |  |  |  |  |
| PSHQ_HW3 | Pearson Correlation | .804^**^ | .719^**^ | .811^**^ | 1 |  |  |  |  |  |  |  |  |
|  | Sig. (2-tailed) | .000 | .000 | .000 |  |  |  |  |  |  |  |  |  |
|  | N | 345 | 345 | 345 | 345 |  |  |  |  |  |  |  |  |
| PSHQ_HW4 | Pearson Correlation | .734^**^ | .518^**^ | .624^**^ | .684^**^ | 1 |  |  |  |  |  |  |  |
|  | Sig. (2-tailed) | .000 | .000 | .000 | .000 |  |  |  |  |  |  |  |  |
|  | N | 345 | 345 | 345 | 345 | 345 |  |  |  |  |  |  |  |
| PSHQ_HW5 | Pearson Correlation | .743^**^ | .502^**^ | .606^**^ | .601^**^ | .616^**^ | 1 |  |  |  |  |  |  |
|  | Sig. (2-tailed) | .000 | .000 | .000 | .000 | .000 |  |  |  |  |  |  |  |
|  | N | 345 | 345 | 345 | 345 | 345 | 345 |  |  |  |  |  |  |
| PSHQ_HW7 | Pearson Correlation | .696^**^ | .456^**^ | .451^**^ | .452^**^ | .384^**^ | .428^**^ | 1 |  |  |  |  |  |
|  | Sig. (2-tailed) | .000 | .000 | .000 | .000 | .000 | .000 |  |  |  |  |  |  |
|  | N | 345 | 345 | 345 | 345 | 345 | 345 | 345 |  |  |  |  |  |
| PSHQ_HW8 | Pearson Correlation | .671^**^ | .479^**^ | .465^**^ | .464^**^ | .389^**^ | .407^**^ | .669^**^ | 1 |  |  |  |  |
|  | Sig. (2-tailed) | .000 | .000 | .000 | .000 | .000 | .000 | .000 |  |  |  |  |  |
|  | N | 345 | 345 | 345 | 345 | 345 | 345 | 345 | 345 |  |  |  |  |
| PSHQ_HW6 | Pearson Correlation | .780^**^ | .534^**^ | .533^**^ | .531^**^ | .443^**^ | .577^**^ | .649^**^ | .560^**^ | 1 |  |  |  |
|  | Sig. (2-tailed) | .000 | .000 | .000 | .000 | .000 | .000 | .000 | .000 |  |  |  |  |
|  | N | 345 | 345 | 345 | 345 | 345 | 345 | 345 | 345 | 345 |  |  |  |
| PSHQ_HW15 | Pearson Correlation | .641^**^ | .309^**^ | .411^**^ | .364^**^ | .435^**^ | .334^**^ | .326^**^ | .263^**^ | .418^**^ | 1 |  |  |
|  | Sig. (2-tailed) | .000 | .000 | .000 | .000 | .000 | .000 | .000 | .000 | .000 |  |  |  |
|  | N | 345 | 345 | 345 | 345 | 345 | 345 | 345 | 345 | 345 | 345 |  |  |
| PSHQ_HW16 | Pearson Correlation | .701^**^ | .347^**^ | .400^**^ | .378^**^ | .372^**^ | .428^**^ | .383^**^ | .373^**^ | .535^**^ | .689^**^ | 1 |  |
|  | Sig. (2-tailed) | .000 | .000 | .000 | .000 | .000 | .000 | .000 | .000 | .000 | .000 |  |  |
|  | N | 345 | 345 | 345 | 345 | 345 | 345 | 345 | 345 | 345 | 345 | 345 |  |
| PSHQ_HW17 | Pearson Correlation | .721^**^ | .391^**^ | .429^**^ | .401^**^ | .379^**^ | .465^**^ | .443^**^ | .367^**^ | .540^**^ | .648^**^ | .793^**^ | 1 |
|  | Sig. (2-tailed) | .000 | .000 | .000 | .000 | .000 | .000 | .000 | .000 | .000 | .000 | .000 |  |
|  | N | 345 | 345 | 345 | 345 | 345 | 345 | 345 | 345 | 345 | 345 | 345 | 345 |
| **. Correlation is significant at the 0.01 level (2-tailed). | | | | | | | | | | | | | |

Sample size = 345, df=343, at α=0.05(95%CI), and two-tailed *rc*​=0.106

1. Experiences

| Correlations | | | | | | | | | | | | | | | | |
| --- | --- | --- | --- | --- | --- | --- | --- | --- | --- | --- | --- | --- | --- | --- | --- | --- |
|  | | ExpTot | SEQ_HW4 | SEQ_HW5 | SEQ_HW8 | SEQ_HW9 | SEQ_HW10 | SEQ_HW11 | SEQ_HW16 | SEQ_HW17 | SEQ_HW18 | SEQ_HW23 | SEQ_HW24 | SEQ_HW26 | SEQ_HW27 | SEQ_HW29 |
| ExpTot | Pearson Correlation | 1 |  |  |  |  |  |  |  |  |  |  |  |  |  |  |
|  | Sig. (2-tailed) |  |  |  |  |  |  |  |  |  |  |  |  |  |  |  |
|  | N | 345 |  |  |  |  |  |  |  |  |  |  |  |  |  |  |
| SEQ_HW4 | Pearson Correlation | .649^**^ | 1 |  |  |  |  |  |  |  |  |  |  |  |  |  |
|  | Sig. (2-tailed) | .000 |  |  |  |  |  |  |  |  |  |  |  |  |  |  |
|  | N | 345 | 345 |  |  |  |  |  |  |  |  |  |  |  |  |  |
| SEQ_HW5 | Pearson Correlation | .701^**^ | .596^**^ | 1 |  |  |  |  |  |  |  |  |  |  |  |  |
|  | Sig. (2-tailed) | .000 | .000 |  |  |  |  |  |  |  |  |  |  |  |  |  |
|  | N | 345 | 345 | 345 |  |  |  |  |  |  |  |  |  |  |  |  |
| SEQ_HW8 | Pearson Correlation | .726^**^ | .540^**^ | .514^**^ | 1 |  |  |  |  |  |  |  |  |  |  |  |
|  | Sig. (2-tailed) | .000 | .000 | .000 |  |  |  |  |  |  |  |  |  |  |  |  |
|  | N | 345 | 345 | 345 | 345 |  |  |  |  |  |  |  |  |  |  |  |
| SEQ_HW9 | Pearson Correlation | .746^**^ | .472^**^ | .566^**^ | .690^**^ | 1 |  |  |  |  |  |  |  |  |  |  |
|  | Sig. (2-tailed) | .000 | .000 | .000 | .000 |  |  |  |  |  |  |  |  |  |  |  |
|  | N | 345 | 345 | 345 | 345 | 345 |  |  |  |  |  |  |  |  |  |  |
| SEQ_HW10 | Pearson Correlation | .722^**^ | .553^**^ | .657^**^ | .562^**^ | .641^**^ | 1 |  |  |  |  |  |  |  |  |  |
|  | Sig. (2-tailed) | .000 | .000 | .000 | .000 | .000 |  |  |  |  |  |  |  |  |  |  |
|  | N | 345 | 345 | 345 | 345 | 345 | 345 |  |  |  |  |  |  |  |  |  |
| SEQ_HW11 | Pearson Correlation | .644^**^ | .351^**^ | .427^**^ | .545^**^ | .558^**^ | .530^**^ | 1 |  |  |  |  |  |  |  |  |
|  | Sig. (2-tailed) | .000 | .000 | .000 | .000 | .000 | .000 |  |  |  |  |  |  |  |  |  |
|  | N | 345 | 345 | 345 | 345 | 345 | 345 | 345 |  |  |  |  |  |  |  |  |
| SEQ_HW16 | Pearson Correlation | .537^**^ | .278^**^ | .215^**^ | .255^**^ | .299^**^ | .261^**^ | .201^**^ | 1 |  |  |  |  |  |  |  |
|  | Sig. (2-tailed) | .000 | .000 | .000 | .000 | .000 | .000 | .000 |  |  |  |  |  |  |  |  |
|  | N | 345 | 345 | 345 | 345 | 345 | 345 | 345 | 345 |  |  |  |  |  |  |  |
| SEQ_HW17 | Pearson Correlation | .589^**^ | .254^**^ | .265^**^ | .353^**^ | .368^**^ | .249^**^ | .353^**^ | .550^**^ | 1 |  |  |  |  |  |  |
|  | Sig. (2-tailed) | .000 | .000 | .000 | .000 | .000 | .000 | .000 | .000 |  |  |  |  |  |  |  |
|  | N | 345 | 345 | 345 | 345 | 345 | 345 | 345 | 345 | 345 |  |  |  |  |  |  |
| SEQ_HW18 | Pearson Correlation | .693^**^ | .330^**^ | .391^**^ | .437^**^ | .423^**^ | .376^**^ | .356^**^ | .448^**^ | .583^**^ | 1 |  |  |  |  |  |
|  | Sig. (2-tailed) | .000 | .000 | .000 | .000 | .000 | .000 | .000 | .000 | .000 |  |  |  |  |  |  |
|  | N | 345 | 345 | 345 | 345 | 345 | 345 | 345 | 345 | 345 | 345 |  |  |  |  |  |
| SEQ_HW23 | Pearson Correlation | .697^**^ | .347^**^ | .389^**^ | .424^**^ | .423^**^ | .412^**^ | .336^**^ | .374^**^ | .324^**^ | .416^**^ | 1 |  |  |  |  |
|  | Sig. (2-tailed) | .000 | .000 | .000 | .000 | .000 | .000 | .000 | .000 | .000 | .000 |  |  |  |  |  |
|  | N | 345 | 345 | 345 | 345 | 345 | 345 | 345 | 345 | 345 | 345 | 345 |  |  |  |  |
| SEQ_HW24 | Pearson Correlation | .698^**^ | .351^**^ | .396^**^ | .373^**^ | .394^**^ | .335^**^ | .299^**^ | .403^**^ | .412^**^ | .498^**^ | .643^**^ | 1 |  |  |  |
|  | Sig. (2-tailed) | .000 | .000 | .000 | .000 | .000 | .000 | .000 | .000 | .000 | .000 | .000 |  |  |  |  |
|  | N | 345 | 345 | 345 | 345 | 345 | 345 | 345 | 345 | 345 | 345 | 345 | 345 |  |  |  |
| SEQ_HW26 | Pearson Correlation | .768^**^ | .409^**^ | .439^**^ | .399^**^ | .452^**^ | .470^**^ | .427^**^ | .343^**^ | .446^**^ | .529^**^ | .567^**^ | .572^**^ | 1 |  |  |
|  | Sig. (2-tailed) | .000 | .000 | .000 | .000 | .000 | .000 | .000 | .000 | .000 | .000 | .000 | .000 |  |  |  |
|  | N | 345 | 345 | 345 | 345 | 345 | 345 | 345 | 345 | 345 | 345 | 345 | 345 | 345 |  |  |
| SEQ_HW27 | Pearson Correlation | .725^**^ | .359^**^ | .428^**^ | .447^**^ | .433^**^ | .391^**^ | .427^**^ | .301^**^ | .348^**^ | .419^**^ | .544^**^ | .558^**^ | .676^**^ | 1 |  |
|  | Sig. (2-tailed) | .000 | .000 | .000 | .000 | .000 | .000 | .000 | .000 | .000 | .000 | .000 | .000 | .000 |  |  |
|  | N | 345 | 345 | 345 | 345 | 345 | 345 | 345 | 345 | 345 | 345 | 345 | 345 | 345 | 345 |  |
| SEQ_HW29 | Pearson Correlation | .660^**^ | .335^**^ | .389^**^ | .343^**^ | .356^**^ | .378^**^ | .301^**^ | .318^**^ | .212^**^ | .447^**^ | .497^**^ | .490^**^ | .621^**^ | .606^**^ | 1 |
|  | Sig. (2-tailed) | .000 | .000 | .000 | .000 | .000 | .000 | .000 | .000 | .000 | .000 | .000 | .000 | .000 | .000 |  |
|  | N | 345 | 345 | 345 | 345 | 345 | 345 | 345 | 345 | 345 | 345 | 345 | 345 | 345 | 345 | 345 |
| **. Correlation is significant at the 0.01 level (2-tailed). | | | | | | | | | | | | | | | | |

1. Coping

| Correlations | | | | | | | | | | | | | | | |
| --- | --- | --- | --- | --- | --- | --- | --- | --- | --- | --- | --- | --- | --- | --- | --- |
|  | | CopTot | SHCQ_HW8 | SHCQ_HW9 | SHCQ_HW10 | SHCQ_HW11 | SHCQ_HW18 | SHCQ_HW19 | SHCQ_HW20 | SHCQ_HW21 | SHCQ_HW22 | SHCQ_HW23 | SHCQ_HW24 | SHCQ_HW25 | SHCQ_HW26 |
| CopTot | Pearson Correlation | 1 |  |  |  |  |  |  |  |  |  |  |  |  |  |
|  | Sig. (2-tailed) |  |  |  |  |  |  |  |  |  |  |  |  |  |  |
|  | N | 345 |  |  |  |  |  |  |  |  |  |  |  |  |  |
| SHCQ_HW8 | Pearson Correlation | .608^**^ | 1 |  |  |  |  |  |  |  |  |  |  |  |  |
|  | Sig. (2-tailed) | .000 |  |  |  |  |  |  |  |  |  |  |  |  |  |
|  | N | 345 | 345 |  |  |  |  |  |  |  |  |  |  |  |  |
| SHCQ_HW9 | Pearson Correlation | .635^**^ | .560^**^ | 1 |  |  |  |  |  |  |  |  |  |  |  |
|  | Sig. (2-tailed) | .000 | .000 |  |  |  |  |  |  |  |  |  |  |  |  |
|  | N | 345 | 345 | 345 |  |  |  |  |  |  |  |  |  |  |  |
| SHCQ_HW10 | Pearson Correlation | .612^**^ | .508^**^ | .519^**^ | 1 |  |  |  |  |  |  |  |  |  |  |
|  | Sig. (2-tailed) | .000 | .000 | .000 |  |  |  |  |  |  |  |  |  |  |  |
|  | N | 345 | 345 | 345 | 345 |  |  |  |  |  |  |  |  |  |  |
| SHCQ_HW11 | Pearson Correlation | .557^**^ | .457^**^ | .537^**^ | .538^**^ | 1 |  |  |  |  |  |  |  |  |  |
|  | Sig. (2-tailed) | .000 | .000 | .000 | .000 |  |  |  |  |  |  |  |  |  |  |
|  | N | 345 | 345 | 345 | 345 | 345 |  |  |  |  |  |  |  |  |  |
| SHCQ_HW18 | Pearson Correlation | .617^**^ | .328^**^ | .314^**^ | .308^**^ | .305^**^ | 1 |  |  |  |  |  |  |  |  |
|  | Sig. (2-tailed) | .000 | .000 | .000 | .000 | .000 |  |  |  |  |  |  |  |  |  |
|  | N | 345 | 345 | 345 | 345 | 345 | 345 |  |  |  |  |  |  |  |  |
| SHCQ_HW19 | Pearson Correlation | .640^**^ | .303^**^ | .306^**^ | .290^**^ | .279^**^ | .701^**^ | 1 |  |  |  |  |  |  |  |
|  | Sig. (2-tailed) | .000 | .000 | .000 | .000 | .000 | .000 |  |  |  |  |  |  |  |  |
|  | N | 345 | 345 | 345 | 345 | 345 | 345 | 345 |  |  |  |  |  |  |  |
| SHCQ_HW20 | Pearson Correlation | .644^**^ | .321^**^ | .265^**^ | .293^**^ | .228^**^ | .582^**^ | .697^**^ | 1 |  |  |  |  |  |  |
|  | Sig. (2-tailed) | .000 | .000 | .000 | .000 | .000 | .000 | .000 |  |  |  |  |  |  |  |
|  | N | 345 | 345 | 345 | 345 | 345 | 345 | 345 | 345 |  |  |  |  |  |  |
| SHCQ_HW21 | Pearson Correlation | .582^**^ | .273^**^ | .256^**^ | .240^**^ | .214^**^ | .410^**^ | .482^**^ | .516^**^ | 1 |  |  |  |  |  |
|  | Sig. (2-tailed) | .000 | .000 | .000 | .000 | .000 | .000 | .000 | .000 |  |  |  |  |  |  |
|  | N | 345 | 345 | 345 | 345 | 345 | 345 | 345 | 345 | 345 |  |  |  |  |  |
| SHCQ_HW22 | Pearson Correlation | .582^**^ | .247^**^ | .285^**^ | .272^**^ | .182^**^ | .097 | .161^**^ | .246^**^ | .326^**^ | 1 |  |  |  |  |
|  | Sig. (2-tailed) | .000 | .000 | .000 | .000 | .001 | .071 | .003 | .000 | .000 |  |  |  |  |  |
|  | N | 345 | 345 | 345 | 345 | 345 | 345 | 345 | 345 | 345 | 345 |  |  |  |  |
| SHCQ_HW23 | Pearson Correlation | .663^**^ | .265^**^ | .309^**^ | .263^**^ | .239^**^ | .259^**^ | .254^**^ | .258^**^ | .295^**^ | .532^**^ | 1 |  |  |  |
|  | Sig. (2-tailed) | .000 | .000 | .000 | .000 | .000 | .000 | .000 | .000 | .000 | .000 |  |  |  |  |
|  | N | 345 | 345 | 345 | 345 | 345 | 345 | 345 | 345 | 345 | 345 | 345 |  |  |  |
| SHCQ_HW24 | Pearson Correlation | .703^**^ | .295^**^ | .359^**^ | .290^**^ | .258^**^ | .319^**^ | .311^**^ | .332^**^ | .227^**^ | .449^**^ | .554^**^ | 1 |  |  |
|  | Sig. (2-tailed) | .000 | .000 | .000 | .000 | .000 | .000 | .000 | .000 | .000 | .000 | .000 |  |  |  |
|  | N | 345 | 345 | 345 | 345 | 345 | 345 | 345 | 345 | 345 | 345 | 345 | 345 |  |  |
| SHCQ_HW25 | Pearson Correlation | .680^**^ | .313^**^ | .346^**^ | .309^**^ | .196^**^ | .186^**^ | .223^**^ | .265^**^ | .245^**^ | .494^**^ | .593^**^ | .663^**^ | 1 |  |
|  | Sig. (2-tailed) | .000 | .000 | .000 | .000 | .000 | .001 | .000 | .000 | .000 | .000 | .000 | .000 |  |  |
|  | N | 345 | 345 | 345 | 345 | 345 | 345 | 345 | 345 | 345 | 345 | 345 | 345 | 345 |  |
| SHCQ_HW26 | Pearson Correlation | .643^**^ | .200^**^ | .206^**^ | .233^**^ | .162^**^ | .333^**^ | .349^**^ | .375^**^ | .318^**^ | .337^**^ | .472^**^ | .561^**^ | .595^**^ | 1 |
|  | Sig. (2-tailed) | .000 | .000 | .000 | .000 | .003 | .000 | .000 | .000 | .000 | .000 | .000 | .000 | .000 |  |
|  | N | 345 | 345 | 345 | 345 | 345 | 345 | 345 | 345 | 345 | 345 | 345 | 345 | 345 | 345 |
| **. Correlation is significant at the 0.01 level (2-tailed). | | | | | | | | | | | | | | | |

SA 7. Predictive Validity

1. Correlation Matrix
2. Perceptions

| Correlation Matrix | | | | | | | | | | | |
| --- | --- | --- | --- | --- | --- | --- | --- | --- | --- | --- | --- |
|  | |  | | **Perceptions** | | **Pressuring** | | **Abducting** | | **Touching** | |
| Perceptions |  | Pearson’s r |  | — |  |  |  |  |  |  |  |
|  |  | p-value |  | — |  |  |  |  |  |  |  |
|  |  | 95% CI Upper |  | — |  |  |  |  |  |  |  |
|  |  | 95% CI Lower |  | — |  |  |  |  |  |  |  |
|  |  | N |  | — |  |  |  |  |  |  |  |
| Pressuring |  | Pearson’s r |  | 0.910 | *** | — |  |  |  |  |  |
|  |  | p-value |  | < .001 |  | — |  |  |  |  |  |
|  |  | 95% CI Upper |  | 0.926 |  | — |  |  |  |  |  |
|  |  | 95% CI Lower |  | 0.890 |  | — |  |  |  |  |  |
|  |  | N |  | 345 |  | — |  |  |  |  |  |
| Abducting |  | Pearson’s r |  | 0.765 | *** | 0.514 | *** | — |  |  |  |
|  |  | p-value |  | < .001 |  | < .001 |  | — |  |  |  |
|  |  | 95% CI Upper |  | 0.806 |  | 0.588 |  | — |  |  |  |
|  |  | 95% CI Lower |  | 0.718 |  | 0.432 |  | — |  |  |  |
|  |  | N |  | 345 |  | 345 |  | — |  |  |  |
| Touching |  | Pearson’s r |  | 0.825 | *** | 0.637 | *** | 0.519 | *** | — |  |
|  |  | p-value |  | < .001 |  | < .001 |  | < .001 |  | — |  |
|  |  | 95% CI Upper |  | 0.856 |  | 0.696 |  | 0.592 |  | — |  |
|  |  | 95% CI Lower |  | 0.788 |  | 0.570 |  | 0.438 |  | — |  |
|  |  | N |  | 345 |  | 345 |  | 345 |  | — |  |
| Note. * p < .05, ** p < .01, *** p < .001 | | | | | | | | | | | |

1. Experiences

|  | |  | | **Experiences** | | **Verbal** | | **Nonverbal** | | **Physical** | |
| --- | --- | --- | --- | --- | --- | --- | --- | --- | --- | --- | --- |
| Experiences |  | Pearson’s r |  | — |  |  |  |  |  |  |  |
|  |  | p-value |  | — |  |  |  |  |  |  |  |
|  |  | 95% CI Upper |  | — |  |  |  |  |  |  |  |
|  |  | 95% CI Lower |  | — |  |  |  |  |  |  |  |
|  |  | N |  | — |  |  |  |  |  |  |  |
| Verbal |  | Pearson’s r |  | 0.885 | *** | — |  |  |  |  |  |
|  |  | p-value |  | < .001 |  | — |  |  |  |  |  |
|  |  | 95% CI Upper |  | 0.906 |  | — |  |  |  |  |  |
|  |  | 95% CI Lower |  | 0.859 |  | — |  |  |  |  |  |
|  |  | N |  | 345 |  | — |  |  |  |  |  |
| Nonverbal |  | Pearson’s r |  | 0.872 | *** | 0.605 | *** | — |  |  |  |
|  |  | p-value |  | < .001 |  | < .001 |  | — |  |  |  |
|  |  | 95% CI Upper |  | 0.895 |  | 0.668 |  | — |  |  |  |
|  |  | 95% CI Lower |  | 0.844 |  | 0.533 |  | — |  |  |  |
|  |  | N |  | 345 |  | 345 |  | — |  |  |  |
| Physical |  | Pearson’s r |  | 0.737 | *** | 0.486 | *** | 0.576 | *** | — |  |
|  |  | p-value |  | < .001 |  | < .001 |  | < .001 |  | — |  |
|  |  | 95% CI Upper |  | 0.781 |  | 0.563 |  | 0.642 |  | — |  |
|  |  | 95% CI Lower |  | 0.684 |  | 0.401 |  | 0.500 |  | — |  |
|  |  | N |  | 345 |  | 345 |  | 345 |  | — |  |
| Note. * p < .05, ** p < .01, *** p < .001 | | | | | | | | | | | |

1. Coping Techniques

|  | |  | | **Coping** | | **Help-seeking** | | **Engagement** | | **Normalisation** | |
| --- | --- | --- | --- | --- | --- | --- | --- | --- | --- | --- | --- |
| Coping |  | Pearson’s r |  | — |  |  |  |  |  |  |  |
|  |  | p-value |  | — |  |  |  |  |  |  |  |
|  |  | 95% CI Upper |  | — |  |  |  |  |  |  |  |
|  |  | 95% CI Lower |  | — |  |  |  |  |  |  |  |
|  |  | N |  | — |  |  |  |  |  |  |  |
| Help-seeking |  | Pearson’s r |  | 0.752 | *** | — |  |  |  |  |  |
|  |  | p-value |  | < .001 |  | — |  |  |  |  |  |
|  |  | 95% CI Upper |  | 0.795 |  | — |  |  |  |  |  |
|  |  | 95% CI Lower |  | 0.703 |  | — |  |  |  |  |  |
|  |  | N |  | 345 |  | — |  |  |  |  |  |
| Engagement |  | Pearson’s r |  | 0.757 | *** | 0.430 | *** | — |  |  |  |
|  |  | p-value |  | < .001 |  | < .001 |  | — |  |  |  |
|  |  | 95% CI Upper |  | 0.799 |  | 0.513 |  | — |  |  |  |
|  |  | 95% CI Lower |  | 0.708 |  | 0.340 |  | — |  |  |  |
|  |  | N |  | 345 |  | 345 |  | — |  |  |  |
| Normalisation |  | Pearson’s r |  | 0.831 | *** | 0.413 | *** | 0.417 | *** | — |  |
|  |  | p-value |  | < .001 |  | < .001 |  | < .001 |  | — |  |
|  |  | 95% CI Upper |  | 0.861 |  | 0.497 |  | 0.501 |  | — |  |
|  |  | 95% CI Lower |  | 0.795 |  | 0.321 |  | 0.326 |  | — |  |
|  |  | N |  | 345 |  | 345 |  | 345 |  | — |  |
| Note. * p < .05, ** p < .01, *** p < .001 | | | | | | | | | | | |

| 1. Measures of predictive Validity for the subscales of sexual harassment perceptions, experiences and coping techniques among Women working in hospitality workplaces of Bahir Dar City (n=345) 2. Perceptions | | | |
| --- | --- | --- | --- |
| **Predictive Validity** | | | |
| Perception Subscales | | | |
| Pressuring | β=9.52, 95% CI: 8.11, 10.94 | | **OR**=1.66, 95% CI: 1.58, 1.74 |
| Touching | β=9.19, 95% CI: 7.07, 11.32 | | **OR**=2.68, 95% CI: 2.48, 2.87 |
| Abducting | β=13.01, 95% CI: 10.74, 15.28 | | **OR**=2.36, 95% CI: 2.15, 2.57 |
| 1. **Experiences** | | | |
| Experiences subscales | |  | |
| Verbal | β=4.19, 95% CI: 3.08, 5.30 | OR=1.73, 95% CI: 1.64, 1.83 | |
| Non-verbal | β=3.89, 95% CI: 2.70, 5.08 | OR=2.07, 95% CI: 1.94, 2.19 | |
| Physical | β=10.63, 95% CI: 9.29, 11.96 | OR=2.99, 95% CI: 2.70, 3.29 | |
| 1. **Coping Techniques** | | | |
| Coping techniques subscales | | |  |
| Help-seeking | β=6.83, 95% CI: 5.34, 8.31 | | OR=2.01, 95% CI: 1.82, 2.19 |
| Engagement | β=11.29, 95% CI: 10.13, 12.44 | | OR=2.00, 95% CI: 1.81, 2.18 |
| Normalisation | β=5.56, 95% CI: 4.32, 6.81 | | OR=1.60, 95% CI: 1.49, 1.72 |
|  | | | |
